# Tailoring water structure with high-tetrahedral-entropy for antifreezing electrolytes and energy storage at −80 °C

**DOI:** 10.1038/s41467-023-36198-5

**Published:** 2023-02-03

**Authors:** Meijia Qiu, Peng Sun, Kai Han, Zhenjiang Pang, Jun Du, Jinliang Li, Jian Chen, Zhong Lin Wang, Wenjie Mai

**Affiliations:** 1 Siyuan Laboratory, Guangzhou Key Laboratory of Vacuum Coating Technologies and New Energy Materials, Guangdong Provincial Engineering Technology Research Center of Vacuum Coating Technologies and New Energy Materials, Department of Physics, Jinan University, Guangzhou, 510632 People’s Republic of China; 2 CAS Center for Excellence in Nanoscience, Beijing Key Laboratory of Micro-Nano Energy and Sensor, Beijing Institute of Nanoenergy and Nanosystems, Chinese Academy of Sciences, Beijing, 100083 People’s Republic of China; 3 Beijing Smart-Chip Microelectronics Technology Co., Ltd, Beijing, 100192 People’s Republic of China; 4 Instrumental Analysis and Research Center, Sun Yat-Sen University, Guangzhou, 510275 People’s Republic of China; 5 School of Materials Science and Engineering, Georgia Institute of Technology, Atlanta, GA 30332 USA; 6 School of Physics, Xidian University, Xi’an, 710071 People’s Republic of China

**Keywords:** Batteries, Batteries, Batteries

## Abstract

One of unsolved puzzles about water lies in how ion-water interplay affects its freezing point. Here, we report the direct link between tetrahedral entropy and the freezing behavior of water in Zn^2+^-based electrolytes by analyzing experimental spectra and molecular simulation results. A higher tetrahedral entropy leads to lower freezing point, and the freezing temperature is directly related to the entropy value. By tailoring the entropy of water using different anions, we develop an ultralow temperature aqueous polyaniline| |Zn battery that exhibits a high capacity (74.17 mAh g^−1^) at 1 A g^−1^ and −80 °C with ~85% capacity retention after 1200 cycles due to the high electrolyte ionic conductivity (1.12 mS cm^−1^). Moreover, an improved cycling life is achieved with ~100% capacity retention after 5000 cycles at −70 °C. The fabricated battery delivers appreciably enhanced performance in terms of frost resistance and stability. This work serves to provide guidance for the design of ultralow temperature aqueous batteries by precisely tuning the water structure within electrolytes.

## Abstract (web summary)

The anti-freezing property of electrolyte is crucial for aqueous batteries under extreme conditions. Here authors explore the relationship between tetrahedral entropy and the freezing behavior of aqueous electrolyte, and further develop anti-freezing electrolyte for aqueous zinc ion batteries.

## Introduction

Despite its simple structure, water is the most important yet least understood liquid on earth^1–7^. The transition from liquid water to solid ice is one of the most common, but complex, whereas vital phenomenon in nature. Undesired ice accumulation can be harmful to human survival and has been linked to aircraft crashes^8^, traffic accidents^9^, tissue/organ inactivation^10^, and battery failure^11–14^. Various strategies for preventing ice formation, including vapor heating, gelation treatment and use of hybrid solvents, have been extensively developed^15–19^. However, these methods are costly, inefficient and can even give rise to environmental pollution. Moreover, the underlying mechanism and driving force for ice nucleation and growth processes remain uncertain^20,21^. The design of an effective antifreeze technique requires a full understanding of the microscopic behavior of water molecules in order to realize practical application, notably the use of antifreezing electrolytes in batteries.

The introduction of various salts into aqueous solution is a simple and low-cost antifreeze strategy. Strong interaction between ions disassociated from dissolved salt solutes and the surrounding water can disrupt the hydrogen bond (HB) networks between water molecules. In addition, the electric field associated with the hydrated ions contribute to the rearrangement of dipolar water molecules. The role of different ions in impacting on the structure of water molecules is the subject of ongoing debate^22–28^. It has been demonstrated that several counterions at the polyelectrolyte brush interface can effectively regulate the dynamics of interfacial water and influence the ice nucleation processes^29^. The specific effects of ions on water structure have been classified into “structure-making” and “structure-breaking”, which is also applied to explain a variety of phenomena in electrolyte systems^30,31^. However, to the best of our knowledge, the thermodynamic origin of how ion specificity determines the freezing point (*T*_*f*_) of water has not been fully understood so far. In the case of batteries operated at ultralow temperatures, antifreezing capability is not the only determining factor; dynamic ion migration of the aqueous electrolyte must be factored in, which is also related to ion-water interaction.

In this study, we utilize tetrahedral entropy^32,33^ (*S*_*Qtet*_, quantifying the distribution of the local tetrahedral order) to bridge the interconnection between salt solutions with different anions and the corresponding antifreeze behavior. Through in-depth studies of the HB structure, kinetic and thermodynamic properties using experimental and theoretical methods, we find that the *S*_*Qtet*_ of water is vital in determining the significant difference in *T*_*f*_ of various salt solutions. The magnitude of *S*_*Qtet*_ is related to both kinetic and thermodynamic properties of the water molecules. Kinetically, faster dynamic behavior such as a higher self-diffusion coefficient and shorter orientation relaxation time facilitates more microscopic states, resulting in a higher *S*_*Qtet*_ within a certain time scale. In terms of thermodynamics, water molecules with higher *S*_*Qtet*_ exhibit lower *T*_*f*_, which can be achieved by tailoring “structure-breaking” ions. As a proof of concept, the cation is fixed and four anions including SO_4_^2-^, Cl^-^, Br^-^ and ClO_4_^-^ are systematically investigated. We find that the “structure-breaking” anion ClO_4_^-^ endows water molecules with the highest *S*_*Qtet*_, and contributes to the best frost resistance. In contrast, “the structure-making” anion SO_4_^2-^delivers the poorest antifreeze ability, originating from its lowest *S*_*Qtet*_ of water. Moreover, we correlate *T*_*f*_ with the tetrahedral entropy in different aqueous salt solutions, confirming the crucial role of *S*_*Qtet*_ in determining water freezing behavior. As validation, we successfully fabricate polyaniline (PANI)||Zn aqueous zinc-ion batteries (ZIBs) utilizing the 5 m Zn(ClO_4_)_2_ electrolyte with a high ionic conductivity (1.12 mS cm^−1^) at −80 °C, which demonstrate enhanced antifreezing performance and stability. A high capacity (74.17 mAh g^−1^) is achieved at 1 A g^−1^ with ~85% capacity retention over 1200 cycles at −80 °C. In addition, 100% capacity is retained after 5000 long-cycles at −70 °C, which can satisfy global extreme cold regions. We also introduce a triboelectric nanogenerator (TENG) to successfully charge the fabricated ZIBs at −80 °C, providing potential application scenario as a self-charging power pack in extreme environments.

## Results

### Guidelines for antifreezing electrolyte

**Fig. 1:**
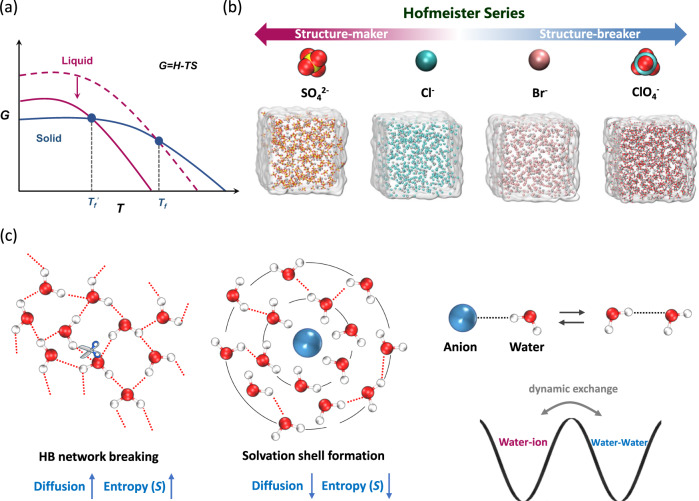
Schematic illustrating the design concept for a low-*T*_*f*_ electrolyte. **a** Relationship between Gibbs free energy, entropy of liquid and the freezing point, in which *S* represents entropy of systems. **b** Hofmeister series with four different anions (top) and the models of the corresponding four aqueous zinc salt solution for MD simulations (bottom). **c** The original water molecules form a tetrahedral network structure through hydrogen bonding. When the network composed of tetrahedrally structured water is disturbed by the introduction of ions, the water diffusion dynamics and *S* increase simultaneously (left). In addition, a solvation shell forms around the ions. As a result of the restriction imposed by the ionic electric field, the diffusion dynamics of water molecules is slowed with a reduction in *S* (middle). Water molecule bonding to an anion and other water molecules serve as two competing dynamic chemical exchange processes (right).

Thermodynamically, the liquidus temperature of electrolytes depends on the Gibbs free energy of solid and liquid (Fig. 1a). In order to lower the *T*_*f*_ of the electrolyte, a decrease in the Gibbs free energy is an essential strategy. According to the thermodynamic equation below,

1 $$G=H-{TS}$$

where *G* (kJ mol^−1^), *H* (kJ mol^−1^), *T* (K) and *S* (J mol^−1^ K^−1^) represent the Gibbs free energy, enthalpy, temperature and entropy of the actual system, respectively. The decrement of Gibbs free energy can be achieved by increasing the entropy of the solvent molecules. Thus, tailoring salty solution with high-entropy water is an effective approach for the design of an antifreezing electrolyte. We selected four salts with different anions (ZnSO_4_, ZnCl_2_, ZnBr_2_ and Zn(ClO_4_)_2_) representing a Hofmeister series for subsequent study (Fig. 1b). The “Hofmeister series” was first proposed to determine the ability of different salts (considering both type and concentration) to stabilize proteins in water. This arrangement is also termed lyotropic sequences or ion specificity series, and has been accepted widely. According to the definition of the series, ions can be classified as structure makers (kosmotropes) and chaos makers (or structure breakers, chaotropes). The former possesses a greater ability to retain order structure, while the latter disrupts the original structure. As shown in Fig. S1a, each water molecule can form four HBs due to the two lone pair electrons on the O atom. Consequently, abundant tetrahedrally structured water molecules occur in pure water. The introduction of a “structure-breaking” salt usually results in a breakdown of the original HB network between water molecules through dipole-dipole interaction (Fig. 1c, left), resulting in a faster diffusion dynamics and higher entropy (*S*). Some water molecules form a solvation shell around ions, restricting movement to some extent with a consequent decrease in *S* (Fig. 1c, middle). Therefore, in salt solutions, water molecules binding to ions or to other water molecules compete in a dynamic chemical exchange process (Fig. 1c, right). Both features should be taken into consideration simultaneously when tailoring the entropy of water.

### Solvation and water structure analysis

**Fig. 2:**
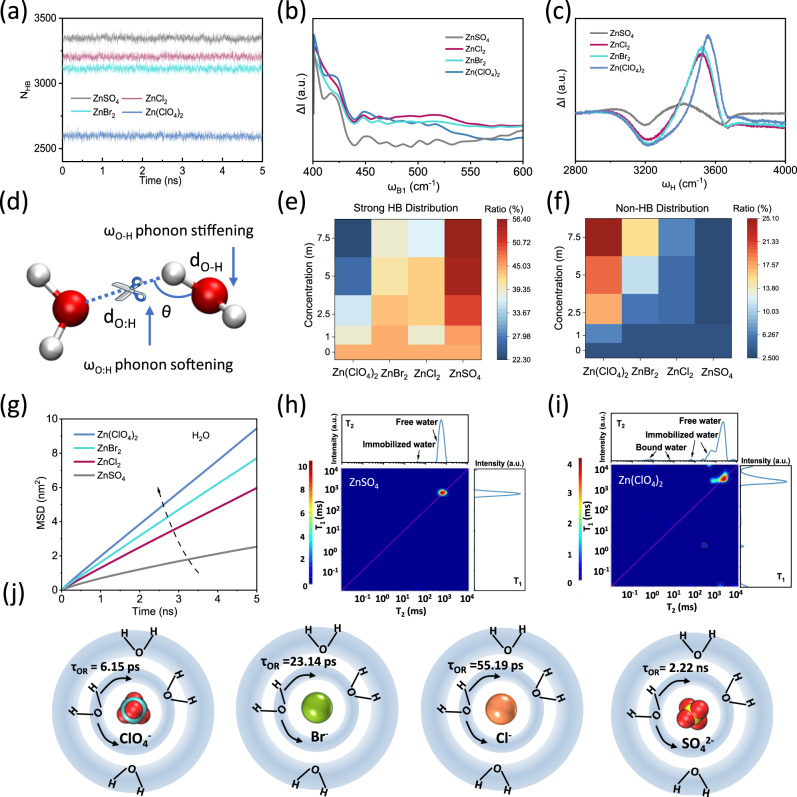
Theoretical and experimental analysis of the HB structure and dynamic relaxation behavior of water. **a** The average HB number of water clusters in four 5 m ZnX (X = ClO_4_^-^, Br^-^, Cl^-^ and SO_4_^2-^) electrolytes collected from MD simulations. Comparison of the DPS of the four electrolytes with respect to the reference spectrum of pure water, which derived from (**b**) FTIR and (**c**) Raman spectra. **d** Schematic showing HB breakage for two water molecules, leading to an elongation of the O:H non-bond and shortening of the O-H polar bond, representing a softening of the O:H phonon and stiffening of the O-H phonon. The proportion of water with strong (**e**) and non-HB (**f**) in four electrolytes at different concentrations fitted from Raman spectra. **g** The simulated MSD versus time curves for water molecules in four electrolytes. 2D LF-NMR T_1_-T_2_ relaxation spectra of (**h**) ZnSO_4_ and (**i**) Zn(ClO_4_)_2_ electrolytes. **j** Schematic showing water structure around ClO_4_^-^, Br^-^, Cl^-^ and SO_4_^2-^. Specified are the time scales associated with orientation relaxation *τ*_*OR*_ of water molecules in the first hydration shell.

As noted above, specific anions impose different impacts on the structure of water in aqueous solution, thus modulating the *S* and determining *T*_*f*_. In order to corroborate our proposed mechanism, classical molecular dynamics (MD) simulations and experimental spectra were subjected to a combined analysis. The average HB number statistics derived from MD simulations for four electrolytes were first performed to assess differences in water structure, where the resultant order (ClO_4_^-^<Br^-^<Cl^-^<SO_4_^2-^) matches the Hofmeister series, as indicated in Fig. 2a. A continuous HB network is difficult to form around the ClO_4_^-^ anion due to its “structure-breaking” characteristics. Fourier transform infrared (FTIR) and Raman spectra were obtained to further confirm the HB statistical results. After background subtraction, FTIR bands (400~600 cm^−1^, *ω*_*B1*_, O:H-O bending vibration mode^34^) and Raman spectra (2800~4000 cm^−1^, *ω*_*H*_, O-H stretching vibration mode^34^) for pure water and the four electrolytes can be normalized with respect to peak area, as shown in Fig. S2a, b. The normalized spectra of the four electrolytes with a subtraction of the pure water component yields differential phonon spectra (DPS). The addition of the four salts all resulted in a red shift of *ω*_*B1*_ and blue shift of *ω*_*H*_ with respect to pure water (the peaks and valleys correspond to phonon abundance gain and loss, respectively). The observed trends indicate that ionic electric-polarization can effectively break the intrinsic HB network, soften the O:H phonon, and stiffen the O-H phonon (Fig. 2b–d). It is found that Δ*ω*_*H*_ decreases in the order ClO_4_^-^>Br^-^>Cl^-^>SO_4_^2-^, establishing the most pronounced effect for ClO_4_^-^ on HB perturbation (Fig. 2c).

To extract more detailed information on HB structure and distribution in different electrolytes, the O-H stretching vibration mode of water was deconvoluted into three Gaussian components:^19^ strong HB (~3230 cm^−1^), weak HB (~3450 cm^−1^) and non-HB (~3620 cm^−1^), as shown in Fig. S3. With an increase in ClO_4_^-^ concentration, the broad peak shifts to a higher frequency with a narrower shape, suggesting that the ratio of strong HB decreases while non-HB increases (Figs. 2e, f and S4a). In contrast, the degree of peak shift with a variation of SO_4_^2-^ electrolyte concentration is relatively minor, implying a negligible enhancement in non-HB (Figs. 2f and S4d). Based on the quantitative comparison of water in different environments (Fig. S4), the HB destruction capability of four anions follows the order ClO_4_^-^>Br^-^>Cl^-^>SO_4_^2-^, in accordance with the MD simulation results. Moreover, the self-diffusion coefficient of water derived from mean square displacement (MSD) curves also follows the sequence ClO_4_^-^>Br^-^>Cl^-^>SO_4_^2-^ (Figs. 2g and S5), which is in good agreement with the data obtained from ^1^H diffusion ordered spectroscopy (DOSY); see Fig. S6. The consistency of these properties can be explained by the more severe disruption of the HB network releasing more free water molecules and facilitating faster self-diffusion.

The 2D low-field nuclear magnetic resonance spectra (LF-NMR) provide relaxation dynamics information for water molecules. The time regions (T_2_, spin-spin relaxation time) in the range 1-10 ms, ~100 ms and ~1000 ms were assigned to bound water, immobilized water and free water, respectively^35,36^. The free water T_1_(spin-lattice relaxation time) was almost equal to T_2_ and located on the diagonal of the 2D T_1_-T_2_ spectra (Fig. S7a). As demonstrated in Figs. 2h, and S7b, c, excess free water is found in SO_4_^2^, Cl^-^ and Br^-^ related electrolytes while the proportion of immobilized and bound water can be neglected, which is similar to that observed for pure water. In contrast, appreciable immobilized and bound water is found in the ClO_4_^-^-based electrolyte (Fig. 2i). Therefore, the HB number between water molecules can be significantly reduced. The HB number between water and the ClO_4_^-^ anion is greater than that of SO_4_^2-^, which can be further confirmed by the HB analysis presented in Fig. S9, and the FTIR measurements in Fig. S10 (23.14 cm^−1^ redshift for ClO_4_^-^ and 16.39 cm^−1^ redshift for SO_4_^2-^). It should be noted that the T_2_ of free water in ClO_4_^-^-based electrolyte is larger than that of SO_4_^2-^, suggesting a faster water molecule relaxation process, consistent with the MSD simulation results. We have also investigated the time scales associated with orientational relaxation (τ_OR_) in the first hydration shells (1HS) of four anions (Figs. 2j and S11). In the case of ClO_4_^-^, the orientational relaxation of water occurs in ~ 6.15 ps, shorter than that of Cl^-^ and Br^-^ (~23.14 ps and 55.19 ps, respectively). Due to the higher valence of SO_4_^2-^, the water molecules are tightly bound and more sluggish (τ_OR_~2.22 ns). Hence, the 1HS of ClO_4_^-^ is lapsable and the inner bound water are freer to rotate, contributing to a higher rotational entropy than the other anions.

To resolve the local structure and diffusion barrier of water molecules in our models involving different anions, radical distribution functions (RDF) and the potential of mean force (PMF) were employed. In Fig. S12a, the RDF of SO_4_^2-^-H (H_2_O) shows a well resolved peak at 1.68 Å associated with 1HS, indicating an ordered distribution of water molecules around SO_4_^2-^. Similar RDFs were generated for Cl^-^ and Br^-^ systems, with peaks located at 2.22 and 2.40 Å, respectively (Fig. S12b, c). On the contrary, the first peak of the ClO_4_^-^-H pair is much weaker and less distinguished (Fig. S12d), indicative of some disorganization within the ClO_4_^-^ solvation shell. The calculated PMF values (derived from RDFs) reflect the kinetic barrier of water molecules in the 1HS, and follow the sequence ClO_4_^-^<Br^-^<Cl^-^<SO_4_^2-^. The relatively weak interaction between ClO_4_^-^ and water molecules leads to facilitated migration and a more disordered distribution of water molecules. Figure S13 illustrates the distribution of the angle *θ* between two vectors, one from the water oxygen atom to the anion and the other bisecting the water molecule. The probability of *θ* in the SO_4_^2-^ electrolyte shows two sharp peaks at around 68° and 120°, while water around ClO_4_^-^ has a broader angle distribution, demonstrating an obvious difference in the degree of disorder, in agreement with the RDF results.

Beside the important function of anions discussed above, the cation (Zn^2+^) owning double charge should be also responsible for the water structure inside the electrolyte system. Therefore, the interaction between Zn^2+^ and H_2_O molecules was studied. Interaction energy of Zn^2+^ with H_2_O inside different systems demonstrate an order of Zn(ClO_4_)_2_ > ZnBr_2_ > ZnCl_2_ > ZnSO_4_ (Fig. S21a), indicating a larger destroying ability to the original water structure. The RDF and corresponding coordination number from MD simulations displaying the distribution situation of the O atom inside H_2_O molecule around the Zn^2+^ were then collected, as shown in Fig. S21b–d. Similar results can be found that Zn^2+^ inside the Zn(ClO_4_)_2_ system possesses the strongest intensity and the largest coordination number with H_2_O compared with other electrolyte systems. The above results can be explained by the weakest interaction between Zn^2+^ and ClO_4_^-^, as exhibited in Fig. S22.

### Solid-liquid transition behavior and tetrahedral entropy analysis

**Fig. 3:**
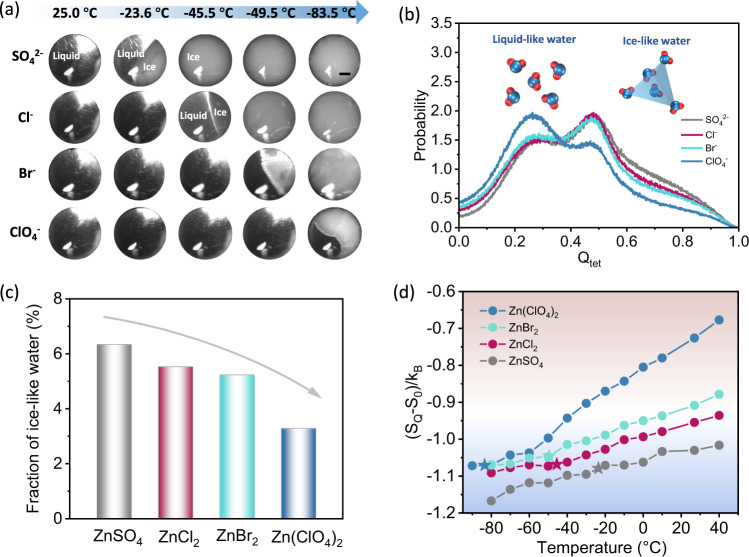
Theoretical and experimental analysis of thermodynamic properties of water in different electrolytes. **a** In situ optical microscopic observations of four electrolytes (5 m) before and after freezing. Black scale bar, 1 mm. **b** The probability distributions of the tetrahedral order parameter *Q*_*tet*_ for water molecules in different electrolytes at 25 °C. Inset shows the schematic models of liquid-like and ice-like water. **c** Fraction of ice-like water (tetrahedrality above 0.8) for four electrolytes at 25 °C. **d** Tetrahedral entropy of water molecules in the four electrolytes, which follows the order SO_4_^2-^ < Cl^-^<Br^-^<ClO_4_^-^ over the temperature range −80 to 40 °C.

Before an in-depth discussion of the different responses in terms of water structure with respect to ion-specificity and the corresponding antifreeze ability, the *T*_*f*_ of different electrolytes should first be determined. We applied an in-situ observation system based on optical microscopy coupled with a digital camera and a cooling stage to monitor the freezing process of different electrolytes (Fig. 3a and S14). As the temperature was lowered from room temperature at a rate of 1 °C min^−1^, water drops (50 μL) containing SO_4_^2-^, Cl^-^, Br^-^ and ClO_4_^-^ with a Zn^2+^ concentration of 5 m (rationale for the choice given in Fig. S15) began to freeze at −23.6, −45.5, −49.5 and −83.5 °C, respectively. The *T*_*f*_ decrease in the anion sequence (ClO_4_^-^<Br^-^<Cl^-^<SO_4_^2-^) matches the previous results. Differential scanning calorimetry (DSC) can determine the thermodynamic change that accompanies temperature (Fig. S16) and the temperature sequence for melting peaks is consistent with the observed *T*_*f*_ above. Figures S17 and S18 show representative snapshots of the ice crystal growing process by MD simulations for pure water and the four electrolytes at different temperatures (−20 and −80 °C). At −20 °C (close to the measured freezing point of −23.5 °C), it should be noted that following sufficient equilibrium relaxation and subsequent 150 ns simulation, the ice crystal growth is obvious and ion diffusion is sluggish in the SO_4_^2-^-based electrolyte (Movie S1). However, the ClO_4_^-^-based electrolyte at the same temperature remains in the liquid state and the ions diffuse uniformly through the entire simulation box (Movie S2). The behavior of Cl^-^ and Br^-^ under the same conditions is similar to ClO_4_^-^. As the temperature was further lowered to −80 °C, only the ClO_4_^-^-based electrolyte system remained in the liquid state, which is consistent with the earlier freezing point results.

The discussion of ion-specificity and freezing point at this juncture has largely drawn on qualitative analysis. To gain greater insight into the underlying mechanism, thermodynamic parameters with quantitative values are required. As noted above, one water molecule forms HBs with another four nearby water molecules, some of which possess quasi-tetrahedral structure. In order to resolve the local water tetrahedral degree, the tetrahedral order parameter (*Q*_*tet*_) can be proposed as^37^:

2 $${Q}_{tet}=1-\frac{3}{8}\mathop{\sum }\limits_{i}^{3}\mathop{\sum }\limits_{j=i+1}^{4}{\left[\cos {\varPsi }_{jik}+\frac{1}{3}\right]}^{2}$$

where *Ψ*_*jik*_ is the angle formed by molecule k and its nearest neighbors i and j. For an ideal tetrahedral structure, this order parameter equals 1 and for ideal gas state, it reduces to 0. Generally, *Q*_*tet*_ of water molecules is distributed between 0 and 1 (Fig. 3b). On this basis, we regard the water molecule with *Q*_*tet*_ above or below 0.8 as ice-like or liquid-like water, respectively. As indicated in Fig. 3c, the fraction of ice-like water in the four electrolytes follows the order of ClO_4_^-^<Br^-^<Cl^-^<SO_4_^2-^, all of which are much lower than that in pure water (Fig. S19). Note that the ice-like water can provide ice nucleation sites, it can be inferred that the ClO_4_^-^-based electrolyte offers the least possibility for promoting the freezing process. Therefore, the freezing point of electrolytes should be strongly related to the water structure (especially *Q*_*tet*_ distribute situation), which is believed to correlate with the entropy of water.

For different aqueous electrolytes, there exist different ratio of tetrahedral structure formed by solely H_2_O, which should be quantified by a more universal parameter. The total entropy of water can be expressed as:

3 $$S={S}_{{id}}+{S}_{{ex}}$$

where *S*_*id*_ is the ideal gas entropy, *S*_*ex*_ is the excess entropy and composed of spatial and orientational distributions in liquid systems. For liquid water, the orientational ordering is vital to decide the total entropy of the system due to the significant variation of the structural ordering of water as a result of the nature of the solute. Kumar et al. have employed the variable *Q*_*tet*_ to define orientational contribution from the total excess entropy that derives from local ordering of water molecules, known as tetrahedral entropy, which can be written as^33^

4 $$\,{S}_{{Q}_{{tet}}}={S}_{0}+\frac{3}{2}{k}_{B}{\int }_{\!\!0}^{1}{{{{{\rm{ln}}}}}}\left(1-{Q}_{{tet}}\right)P\left({Q}_{{tet}}\right)d{Q}_{{tet}}$$

where *S*_0_ is a constant, *k*_*B*_ is the Boltzmann constant, *P* (*Q*_*tet*_) represents the distribution of *Q*_*tet*_ at a given temperature *T*. It can be used to describe the deviation degree of the total *S* of the system from ideal tetrahedral structure of the water molecule network. With a more negative ${S}_{{Q}_{tet}}$, the severer deviation of the average water distribution from tetrahedral structure.

The tetrahedral entropy ${S}_{{Q}_{tet}}$ was calculated using Eq. (4) based on the population of the tetrahedral order parameter ${Q}_{tet}$ in different systems. Obviously, the pure water exhibits the lowest tetrahedral entropy at room temperature [$({S}_{{Q}_{tet}}-{S}_{0})/{k}_{B}=-1.770$, Table S1], and the order of ${S}_{{Q}_{tet}}$ in the four electrolytes follows the sequence of ClO_4_^-^>Br^-^>Cl^-^>SO_4_^2-^, in accordance with the population of ice-like water shown in Fig. 3c. The sequence remains unchanged at different simulated temperatures ranging from −80 to 40 °C (Fig. 3d). Strikingly, the *T*_*f*_ of water derived from experiments in different electrolytes resulted in very similar ${S}_{{Q}_{tet}}$ (denoted by star symbols in Fig. 3d), which we proposed as a threshold value. The traditional colligative view ignores an ion-specific effect and fails to explain why different electrolytes (at the same concentration) often exhibit distinct frost resistance. The ${S}_{{Q}_{tet}}$parameter provides a new understanding at a micro-scale that better accounts for and predicts the solid-liquid transition of various electrolytes. Three additional Zn salts with different anions (Zn(TFSI)_2_, ZnI_2_ and Zn(NO_3_)_2_) were further introduced to verify the reliability of the above proposal. Combining both experimental observation (determining the freezing points as −40.0 °C, −43.0 °C and −62.0 °C for Zn(TFSI)_2_, Zn(NO_3_)_2_ and ZnI_2_, respectively) and theorical calculation, the ${S}_{{Q}_{tet}}$for Zn(TFSI)_2_, ZnI_2_ and Zn(NO_3_)_2_ at each individual *T*_*f*_ generated values close to those obtained for the previous four Zn salts (Figs. S23 and S24). These results validate the use of the threshold value of ${S}_{{Q}_{tet}}$ to predict *T*_*f*_. Both MD simulations and experimental results confirm that differences in *T*_*f*_ of different salt solutions with unique anions are strongly related to the ${S}_{{Q}_{tet}}$ of water molecules.

The concentration of electrolyte is an important factor related to the *T*_*f*_, which should be studied further. The most proper concentration of 5 m for Zn(ClO_4_)_2_ was sifted according to the DSC results, as presented in Fig. S15. However, the variation of concentration will greatly determine the number of free H_2_O molecules and also the proportion of ice-like water even at their *T*_*f*_ (Fig. S25). Therefore, it is hard to judge the *T*_*f*_ of any Zn-based electrolyte with random concentration *via* the ${S}_{{Q}_{tet}}$ value. To verify the versatility of the ${S}_{{Q}_{tet}}$ value for judging the *T*_*f*_ of different Zn-based electrolytes with a same concentration, DSC experiments and MD simulation were further conducted for 1 m and 3 m samples. It can be found that the *T*_*f*_ value of several electrolytes with different anions overall follow well with the order similar to the situation of 5 m, as exhibited in Fig. S26. The ${S}_{{Q}_{tet}}$ under the above *T*_*f*_ were further calculated, as indicated in Fig. S27. Rather close values can both be achieved under the concentration of 1 m and 3 m with different electrolytes, solidly confirming the universality of this judging strategy for Zn-based electrolyte owning different anions but the same concentration.

### Low-temperature performance of PANI||Zn batteries

**Fig. 4:**
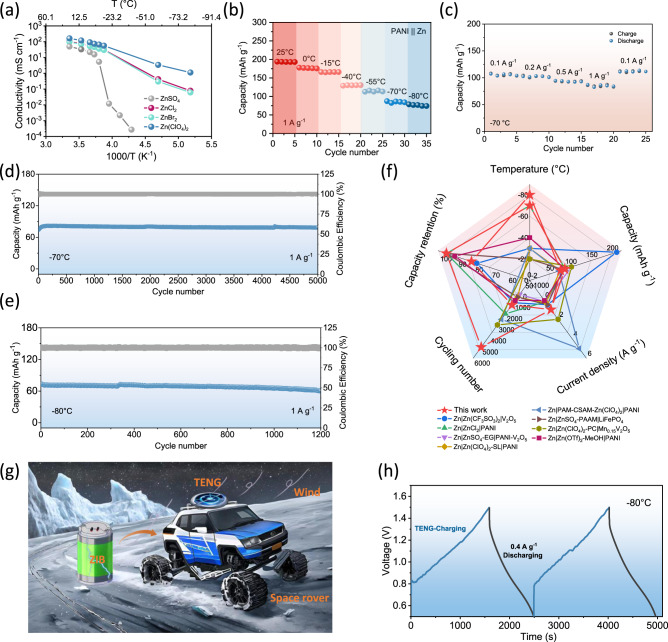
The electrochemical performance of PANI||Zn full batteries and self-power systems at different temperatures. **a** Ionic conductivities of the four electrolytes over the temperature range of −80~+25 °C. **b** The capacity and charge-discharge curves of PANI||Zn full batteries at 1 A g^−1^ and temperatures in the range 25 °C to −80 °C. **c** Rate performance at different current densities (0.1 to 1 A g^−1^) at −70 °C. Cycling performance (1 A g^−1^) at (**d**) −70 °C and (**e**) −80 °C. **f** Low-temperature performance comparison of this work with previously reported ZIB systems. **g** Schematic illustrating the self-powered system combining ZIB and TENG for powering a space rover under extremely cold conditions. **h** TENG-charge and galvanostatic discharge performance of the self-powered system at −80 °C.

Due to the outstanding antifreeze capability exhibited by the ClO_4_^-^-based electrolyte, it has been identified as a competitive candidate for practical applications in low-temperature batteries. Apart from preventing electrolyte freezing, high ionic conduction at low-temperature should also be taken into account. In the case of 5 m Zn(ClO_4_)_2_ electrolyte, liquid-like water predominates with less HB due to water-water interaction when compared with ZnSO_4_, ZnCl_2_ and ZnBr_2_. In Zn(ClO_4_)_2_, the lesser limitation from both the ion solvation shell and bulk electrolyte environment permits greater migration of anions and cations. The ionic conductivities curves of the four standard electrolytes (5 m ZnX, X = SO_4_^2-^, Cl^-^, Br^-^, ClO_4_^-^) and one additional electrolyte (ZnTFSI) over the temperature range −80~+25 °C are shown in Figs. 4a and S28. The ZnSO_4_ electrolyte exhibits a fast decay of ionic conductivity at −20 °C due to freezing. The decay speed varied at lower temperatures for Zn(TFSI)_2_ with an abrupt decrease at −40 °C. By comparison, Zn(ClO_4_)_2_ exhibited the highest ionic conductivity over −80~+25 °C, which agrees with the simulated MSD results and calculated self-diffusion coefficients (Fig. S29). Owing to the lower solid-liquid transition temperature, the 5 m Zn(ClO_4_)_2_ electrolyte exhibited sufficient ionic conductivity (1.12 mS cm^−1^) at the ultralow temperature (−80 °C). This can be ascribed to weak cation interaction with ClO_4_^-^, resulting in a dispersed solvation structure, as shown in Fig. S22. The weak coupling of cation and anion affords more free ions to migrate, which guarantees high conductivity. Taking an overview of the reported electrolyte systems with remarkable anti-freezing ability^19,38–41^, the 5 m Zn(ClO_4_)_2_ used in this work outperforms all others, notably at the ultra-low temperature of −80 °C (Fig. S30). This unique property brings great performance of full cells under an extreme low-temperature environment.

Since the performance of the full cell is mainly determined by the cathode, it is essential to choose a candidate with sufficient capacity even under ultralow temperature conditions. For traditional inorganic cathodes including Mn, V and Prussian blue (or analogues) based-materials, sluggish Zn^2+^ kinetics can be attributed to the high associated charge, especially at low temperatures. Consequently, organic materials with charge storage capability involving surface coordination with ions have been studied intensively for use in low temperature batteries^19,42–44^. PANI is a commonly used cathode for ZIBs that can store/release Zn^2+^ through dynamic coupling/decoupling processes, as illustrated in Fig. S31. In theory, PANI can retain fast ion kinetics even at low temperatures and was accordingly chosen in this study. The capacity and charge-discharge curves of PANI||Zn full batteries utilizing Zn(ClO_4_)_2_ as electrolyte at 1 A g^−1^ and varying temperature are shown in Fig. 4b and S32a. The system exhibited remarkable low-temperature tolerance and high-capacity retention (67.5%) at −40 °C. Three fabricated PANI||Zn pouch cells trapped in ice and operated in series can successfully power LED sets (Fig. S32b). Even at the ultralow temperature of −80 °C, the battery retained a high capacity (74.17 mAh g^−1^) at 1 A g^−1^ (Fig. 4b). Fig. 4c demonstrates excellent rate performance ranging from 0.1 A g^−1^ to 1 A g^−1^ at −70 °C. Ultra-stable cycling performance over 5000 cycles with ~100% capacity retention and over 1200 cycles with ~85% capacity retention were achieved at −70 °C and −80 °C, respectively (Fig. 4d, e). This level of performance can satisfy the demand for large-scale application under extremely cold conditions, such as polar regions and in space exploration. The performance achieved in this study in terms of working temperature, current density, capacity and cycling stability is compared with existing ZIBs^15,19,36,45–49^ and other batteries^21,50–56^ in Figs. 4f and S33, respectively. Our fabricated ZIBs delivered the best low temperature performance with respect to frost resistance and cycling stability, exhibiting enormous potential in extreme environments. The schematic shown in Fig. 4g illustrates a potential future application scenario for TENG charged antifreezing ZIBs. The TENG can be mounted on top of a space rover and harvest mechanical energy from natural wind in other planets to charge our special ZIBs to supply more stored power under extreme low temperatures. As validation, one ZIB was charged by TENG^20^ and discharged at a constant current density of 0.4 A g^−1^ at −80 °C for 2 cycles with satisfactory results (Figs. S34 and 4h). This demonstrates the feasibility of constructing this self-charging power pack with operation in an extremely cold environment.

## Discussion

In this work, we quantitatively related an entropic contribution to the *T*_*f*_ of water molecules from a consideration of ion-specific effects. Through in-depth and systematic studies combining multiple experimental methods and theoretical simulations, the HB structure and critical kinetic and thermodynamic data for water molecules in different electrolytes are determined. The water from the ClO_4_^-^-based electrolyte system with intrinsically higher tetrahedral entropy exhibits the best frost resistance. The *T*_*f*_ of water molecules in different electrolytes can be unified on the basis of a common tetrahedral entropy threshold region. The results demonstrate the crucial role played by tetrahedral entropy and provides a novel approach to understanding water freezing behavior. Guided by this theory, we developed an ultralow temperature aqueous PANI||Zn battery (with a high electrolyte ionic conductivity of 1.12 mS cm^−1^ at −80 °C), exhibiting a high capacity (74.17 mAh g^−1^) at −80 °C and 1 A g^−1^ with a capacity retention of ~85% after 1200 cycles. Moreover, a much better cycling life of 5000 repeats with ~100% capacity retention at −70 °C has also been achieved. Our fabricated battery can be charged using a TENG at an ultralow temperature (−80 °C) and discharged with a stable rate of 0.4 A g^−1^, confirming feasible application as a self-powered system under extreme conditions. The principles that underpin this work can inform future exploration of the thermodynamic fundamentals of phase transition in designing next generation antifreezing electrolytes for practical application.

## Methods

### Materials

Zn foils (>99.99%) of 100 μm were used for full batteries construction. PANI was grown on carbon paper by ammonium persulfate oxidizing aniline monomer in aqueous acid^57^. First, 3.65 mL aniline monomer was added into 150 mL HCl (1 M), then carbon papers were soaked in the solution and keep stirring for 1 h. After that, 150 mL 1 M HCl containing 2.28 g ammonium persulfate was poured into above solution under an ice bath, and the mixed solution was keep stirring for another 1 h. Finally, the carbon papers with dark green PANI were taken out, washed with deionized water and dried at 60 °C for 8 h. The mass loading of the PANI is around 1.1 mg cm^−2^.

### Fabrication of the ZIBs

Commercial Zn sheets (with a thickness of 100 μm and diameter of 10 mm) were utilized as anodes. Before the fabrication, Zn sheets were washed by acetone, ethanol and deionized water in succession. One pierce of Zn sheet anode and PANI cathode were used as two electrodes for constructing the ZIB. 75 μL 5 m Zn(ClO_4_)_2_ electrolyte were added into the CR2032 coin cell separated by a piece of glass fiber (GE-Whatman, 125 mm).

### Fabrication of the TENG

The rotor is an epoxy glass fiber disk board which has sixteen pieces of 80 μm thick fan-shaped fluorinated ethylene-propylene (FEP) copolymer film stuck on it. The thickness of the rotor is 0.3 mm and the diameter is 20 cm. The stator is a custom-made printed circuit board (PCB) which has 16 pairs of copper (Cu) electrodes. There is a small vertical gap (<0.3 mm) between the rotor and the stator. Two stripes of artificial wool cloth with a width of 2 cm were stuck on a PMMA disk substrate to make triboelectrification brushes.

### Simulation methods

Molecular dynamics simulations were performed with GROMACS^58^ both in the isothermal-isochoric (NVT) and the isothermal isobaric (NPT) ensembles. In both cases, a leap frog integrator with a time step of 1 fs was used. A 1.0 nm cutoff distance were used for the van der Waals interactions and Electrostatic interactions. Electrostatic interactions were calculated using the particle mesh Ewald method^59^ (PME). In the NVT simulations, V-rescale^60^ thermostat with a characteristic time of 2 ns implemented the constant temperature condition. In the NPT simulations, 1 atm pressure was maintained via an isotropic Parrinello Rahman barostat. For simulations without ice slab, the dimension of the cubic box (with periodic boundary conditions) is 4.5 × 4.5 × 4.5 nm^3^, consisting 3025 water molecules, 275 Zn^2+^ and 275 anions (for SO_4_^2-^) or 550 anions (for Cl^-^, Br or ClO_4_^-^). The OPC3^61^ water model was adopted, and the interaction parameters for ions were taken from GAFF and Merz^62^ force field.

The MD simulation for different electrolyte systems from 243 K to 313 K at 1 atm were performed for 60 ns to ensure reaching equilibrium, and the last 10 ns simulation in the equilibrium state were used for analysis. The calculation of hydrogen bond (HB) is based on the geometrical configuration that the distance of two O is <3.5 Å and the angle of O–H···O is <30°. For simulations with initial ice slab, the dimension of the simulation box (with periodic boundary conditions) is 2.8 × 2.3 × 6.9 nm^3^. The initial ice slab consisted 864 water molecules and the liquid slab contained 600 water molecules, 55 Zn^2+^ and 55 anions (for SO_4_^2-^) or 110 anions (for Cl^-^, Br^-^ or ClO_4_^-^). The TIP4P/ICE water model was used in this two-phase system. This water model is especially well suited to perform MD simulations with ice crystal^63^. The parameters of ions were the same as those above. The two-phase systems were first equilibrated at NVT (using V-rescale thermostat for 1 ns) and NPT ensemble (using Parrinello-Rahman barostat for 1 ns), and then a 150 ns production run at 253 K were conducted.

### Potential of mean force (PMF) analysis

The PMF is related to the RDF using the equation PMF(r) = −RTln(g(r)), which reflects the kinetic barrier of water molecules in the first solvation shell.

### Mean square displacement (MSD) analysis

MSD measures the mean of the square of particle displacement over time t. *MSD*(*t*) = <|*r*(*t*)−*r*(0)|^2^>.

### Self-diffusion coefficient *D* of water molecules

The self-diffusion coefficient *D* of water molecules was derived according to the ${{{{{\rm{Einstein}}}}}}\,{{{{{\rm{equation}}}}}}\,D=\frac{1}{6}{{{{{\mathrm{lim}}}}}}_{t\to \infty }\frac{MSD(t)}{t}.$

### Orientational relaxation time τ_OR_ of water in first solvation shell of anions

The mean orientational relaxation time of water in first solvation shell of anions are calculated by using the autocorrelation function of orientational order parameter

<*P*_2_ (*t*) *P*_2_ (0)>, where ${P}_{2}(t)=\frac{1}{2}(3{{{\cos }}}^{2}\left(\delta \theta \left(t\right)\right)-1).\delta \theta \left(t\right)=\theta \left(t\right)-\theta \left(0\right)$ represents the angle between dipole vectors of a water molecule at times t and 0.

### Characterizations

DSC was carried out in METTLER TOLEDD DSC3 in the procedure of +20~−80 °C with a cooling rate of 5 °C min^−1^, constant temperature for 2 mins and −80~+20 °C with a heating rate of 5 °C min^−1^. Raman spectroscopy was conducted on Horiba LabRAM HR Evolution microscope and a 532 nm excitation laser was used. FTIR spectroscopy was carried out on FTIR-850 (Tianjin Gangdong Sci.&Tech). The LF-NMR analyzer (NMI20-030H-I, Shanghai Niumag Corporation, Shanghai, China) was used in the measurements of 2D T_1_-T_2_ relaxation. The in-situ microscope images of freezing process were obtained by a commercial high-resolution digital camera (Mshot, MS60) equipped with a cooling stage (Linkam THMS600). A direct current motor (XCWS57-30W, WCDQ) was used as the simulated mechanical energy source. The basic performance of TENG was measured by an electrometer (6514, Keithley) and an oscilloscope (MDO3104, Tektronix) with a high-voltage probe (500 MΩ).

### Electrochemical measurements

The rate performance and cycling stability of ZIBs were conducted by the Neware battery test system (CT-4008Tn-5V10mA-164, Shenzhen, China). The cutoff voltages were set as 0.5 V and 1.5 V, separately for all electrochemical tests. For rate performance, different current densities of 0.1, 0.2, 0.5 and 1 A g^−1^ were used while for long-term cycling test, 1 A g^−1^ were chosen. The discharge–curves of the TENG-ZIBs system were conducted by CHI660E. All low-temperature performances were conducted in HARCICRY refrigerators (Q/22, −15~25 °C) and Jiesheng ultra-low temperature refrigerators (DW-86W28, −86~−40 °C).

## Supplementary information

Supplementary Information

Description of Additional Supplementary Files

Supplementary Movie 1

Supplementary Movie 2

## Data Availability

The data that support the findings of this study are available from the corresponding author upon reasonable request.

## Code availability

The codes that support the findings of this study are available from the corresponding author upon reasonable request.

## References

[1] Xu L (2009). Appearance of a fractional Stokes–Einstein relation in water and a structural interpretation of its onset. Nat. Phys..

[2] Qi C, Zhu Z, Wang C, Zheng Y (2021). Anomalously low dielectric constant of ordered interfacial water. J. Phys. Chem. Lett..

[3] Moore EB, Molinero V (2011). Structural transformation in supercooled water controls the crystallization rate of ice. Nature.

[4] Ben-Amotz D (2022). Electric buzz in a glass of pure water. Science.

[5] Wang YH (2021). In situ Raman spectroscopy reveals the structure and dissociation of interfacial water. Nature.

[6] Pullanchery S, Kulik S, Rehl B, Hassanali A, Roke S (2021). Charge transfer across C–H⋅⋅⋅O hydrogen bonds stabilizes oil droplets in water. Science.

[7] Yang J (2021). Direct observation of ultrafast hydrogen bond strengthening in liquid water. Nature.

[8] Marwitz J (1997). Meteorological conditions associated with the ATR72 Aircraft Accident near Roselawn, Indiana, on 31 October 1994. B. Am. Meteorol. Soc..

[9] Andersson AK, Chapman L (2011). The impact of climate change on winter road maintenance and traffic accidents in West Midlands, UK. Accid. Anal. Prev..

[10] Tessier SN (2018). Effect of ice nucleation and cryoprotectants during high subzero-preservation in endothelialized microchannels. ACS Biomater. Sci. Eng..

[11] Sun Y (2021). Salty ice electrolyte with superior ionic conductivity towards low‐temperature aqueous zinc ion hybrid capacitors. Adv. Funct. Mater..

[12] Zhao CX (2021). Can aqueous zinc-air batteries work at sub-zero temperatures?. Angew. Chem. Int. Ed..

[13] Nian Q (2019). Aqueous batteries operated at −50 °C. Angew. Chem. Int. Ed..

[14] Feng Y (2022). Challenges and advances in wide-temperature rechargeable lithium batteries. Energy Environ. Sci..

[15] Chang N (2020). An aqueous hybrid electrolyte for low-temperature zinc-based energy storage devices. Energy Environ. Sci..

[16] Lv J, Song Y, Jiang L, Wang J (2014). Bio-inspired strategies for anti-icing. ACS Nano.

[17] He Z (2020). Bioinspired multifunctional anti-icing hydrogel. Matter.

[18] Xu X, Jerca VV, Hoogenboom R (2020). Bio-inspired hydrogels as multi-task anti-icing hydrogel. Coat. Chem..

[19] Zhang Q (2020). Modulating electrolyte structure for ultralow temperature aqueous zinc batteries. Nat. Commun..

[20] Hofhuis K (2022). Real-space imaging of phase transitions in bridged artificial kagome spin ice. Nat. Phys..

[21] Jiang H (2020). A high‐rate aqueous proton battery delivering power below −78 °C via an unfrozen phosphoric acid. Adv. Energy Mater..

[22] Tielrooij KJ, Garcia-Araez N, Bonn M, Bakker HJ (2010). Cooperativity in ion hydration. Science.

[23] Tobias DJ, Hemminger JCChemistry (2008). Getting specific about specific ion effects. Science.

[24] Wu S (2017). Ion-specific ice recrystallization provides a facile approach for the fabrication of porous materials. Nat. Commun..

[25] Lee Y, Thirumalai D, Hyeon C (2017). Ultrasensitivity of water exchange kinetics to the size of metal ion. J. Am. Chem. Soc..

[26] Foglia F (2022). Disentangling water, ion and polymer dynamics in an anion exchange membrane. Nat. Mater..

[27] Zhang C, Yue S, Panagiotopoulos AZ, Klein ML, Wu X (2022). Dissolving salt is not equivalent to applying a pressure on water. Nat. Commun..

[28] Guo J (2020). Hydration of NH_4_^+^ in Water: Bifurcated Hydrogen Bonding Structures and Fast Rotational Dynamics. Phys. Rev. Lett..

[29] He Z (2016). Tuning ice nucleation with counterions on polyelectrolyte brush surfaces. Sci. Adv..

[30] Marcus Y (2009). Effect of ions on the structure of water: structure making and breaking. Chem. Rev..

[31] Ji X (2021). A perspective of ZnCl_2_ electrolytes: The physical and electrochemical properties. eScience.

[32] Banerjee P, Bagchi B (2020). Role of local order in anomalous ion diffusion: Interrogation through tetrahedral entropy of aqueous solvation shells. J. Chem. Phys..

[33] Kumar P, Buldyrev SV, Stanley HE (2009). A tetrahedral entropy for water. Proc. Natl Acad. Sci..

[34] Zhang X (2014). Mediating relaxation and polarization of hydrogen-bonds in water by NaCl salting and heating. Phys. Chem. Chem. Phys..

[35] Wang S, Lin R, Cheng S, Tan M (2020). Water dynamics changes and protein denaturation in surf clam evaluated by two-dimensional LF-NMR T1-T2 relaxation technique during heating process. Food Chem..

[36] Huang S, Hou L, Li T, Jiao Y, Wu P (2022). Antifreezing hydrogel electrolyte with ternary hydrogen bonding for high-performance zinc-ion batteries. Adv. Mater..

[37] Errington JR, Debenedetti PG (2001). Relationship between structural order and the anomalies of liquid water. Nature.

[38] Chen S, Peng C, Xue D, Ma L, Zhi C (2022). Alkaline tolerant antifreezing additive enabling aqueous Zn||Ni battery operating at −60 °C. Angew. Chem. Int. Ed..

[39] Nian Q (2019). Aqueous batteries operated at −50 °C. Angew. Chem. Int. Ed..

[40] Sun T (2021). An ultralow-temperature aqueous zinc-ion battery. J. Mater. Chem. A.

[41] Sun T, Zheng S, Du H, Tao Z (2021). Synergistic effect of cation and anion for low-temperature aqueous zinc-ion battery. Nano Micro Lett..

[42] Dong X, Guo Z, Guo Z, Wang Y, Xia Y (2018). Organic batteries operated at −70 °C. Joule.

[43] Dong X (2019). High‐energy rechargeable metallic lithium battery at −70 °C enabled by a cosolvent electrolyte. Angew. Chem. Int. Ed..

[44] Liang Y (2017). Universal quinone electrodes for long cycle life aqueous rechargeable batteries. Nat. Mater..

[45] Geng H (2019). Electronic structure regulation of layered vanadium oxide via interlayer doping strategy toward superior high‐rate and low‐temperature zinc‐ion batteries. Adv. Funct. Mater..

[46] Lin X (2021). Hydrated deep eutectic electrolytes for high‐performance Zn‐Ion batteries capable of low‐temperature operation. Adv. Funct. Mater..

[47] Zhang Q (2021). Chaotropic anion and fast-kinetics cathode enabling low-temperature aqueous Zn batteries. ACS Energy Lett..

[48] Zhu M (2019). Antifreezing hydrogel with high zinc reversibility for flexible and durable aqueous batteries by cooperative hydrated cations. Adv. Funct. Mater..

[49] Ma L (2022). Highly reversible Zn metal anode enabled by sustainable hydroxyl chemistry. Proc. Natl Acad. Sci..

[50] Liang H (2022). Unusual mesoporous titanium niobium oxides realizing sodium-ion batteries operated at −40 °C. Adv. Mater..

[51] Rustomji CS (2017). Liquefied gas electrolytes for electrochemical energy storage devices. Science.

[52] Xu J (2019). Extending the low temperature operational limit of Li-ion battery to −80 °C. Energy Storage Mater..

[53] Yue F (2021). An ultralow temperature aqueous battery with proton chemistry. Angew. Chem. Int. Ed..

[54] Zhang W (2021). Decimal solvent-based high-entropy electrolyte enabling the extended survival temperature of Lithium-Ion Batteries to −130 °C. CCS Chem..

[55] Zhu K (2022). Inorganic electrolyte for low-temperature aqueous sodium ion batteries. Small.

[56] Zhu Y (2022). Hydrated eutectic electrolytes for high-performance Mg-ion batteries. Energy Environ. Sci..

[57] Wan F (2018). An aqueous rechargeable zinc-organic battery with hybrid mechanism. Adv. Funct. Mater..

[58] Berendsen HJC, van der Spoel D, van Drunen R (1995). GROMACS: A message-passing parallel molecular dynamics implementation. Comput. Phys. Commun..

[59] Gingrich TR, Wilson M (2010). On the Ewald summation of Gaussian charges for the simulation of metallic surfaces. Chem. Phys. Lett..

[60] Bussi G, Donadio D, Parrinello M (2007). Canonical sampling through velocity rescaling. J. Chem. Phys..

[61] Izadi S, Onufriev AV (2016). Accuracy limit of rigid 3-point water models. J. Chem. Phys..

[62] Li Z, Song LF, Li P, Merz KM (2020). Systematic parametrization of divalent metal ions for the OPC3, OPC, TIP3P-FB, and TIP4P-FB water models. J. Chem. Theory Comput..

[63] Abascal JL, Sanz E, Garcia Fernandez R, Vega C (2005). A potential model for the study of ices and amorphous water: TIP4P/Ice. J. Chem. Phys..

