# Supplementary material for: Tailoring water structure with high-tetrahedral-entropy for antifreezing electrolytes and energy storage at −80 °C
Source: Nat Commun. 2023 Feb 3;14:601. doi: 10.1038/s41467-023-36198-5 (PMC9898254; doi:10.1038/s41467-023-36198-5)
Supplement: Supplementary file 1 — Supplementary Information [file 41467_2023_36198_MOESM1_ESM.pdf]

Supplementary Materials for

Tailoring water structure with high-tetrahedral-entropy for antifreezing electrolytes and energy storage at -80 °C

Meijia Qiu[1], Peng Sun[1], Kai Han[1, 2], Zhenjiang Pang[3], Jun Du[3], Jinliang Li[1], Jian Chen[4], Zhong Lin Wang[2, 5,*], Wenjie Mai[1, 2,*]

[1] Siyuan Laboratory, Guangzhou Key Laboratory of Vacuum Coating Technologies and New Energy Materials, Guangdong Provincial Engineering Technology Research Center of Vacuum Coating Technologies and New Energy Materials, Department of Physics, Jinan University, Guangdong 510632, People's Republic of China

[2] CAS Center for Excellence in Nanoscience, Beijing Key Laboratory of Micro-Nano Energy and Sensor, Beijing Institute of Nanoenergy and Nanosystems, Chinese Academy of Sciences, Beijing 100083, People's Republic of China

[3] Beijing Smart-Chip Microelectronics Technology Co., Ltd., Beijing, 100192, People's Republic of China

[4] Instrumental Analysis and Research Center, Sun Yat-Sen University, Guangzhou 510275, People's Republic of China

[5] School of Materials Science and Engineering, Georgia Institute of Technology, Atlanta, GA 30332, USA

*Corresponding authors: zhong.wang@mse.gatech.edu (ZLW); wenjiemai@email.jnu.edu.cn (WJM)

Contents

Supplementary Figures 4

Figure S1 (a) The electronic structure of water molecule. (b)The proportion of water molecules without or with one HB in pure water and four electrolytes at 300K. (c)The HB autocorrelation function C(t) and (d) HB lifetime between water molecules in four electrolytes at 300K. 4

Figure S2 (a) FTIR spectra in the range of 400~600 cm^{-1} and (b)Raman spectra in the range of 2800~4000 cm^{-1}. 5

Figure S3 Raman spectroscopy of O–H stretching vibration in pure water, which can be divided into three parts (water molecules with strong HB, weak HB and non-HB). 6

Figure S4 (a-d) Raman spectroscopy of O–H stretching vibration in four electrolytes with different concentrations. (e-h) The proportion of water with strong HB, weak HB and non-HB in four electrolytes with different concentrations. 7

Figure S5 Self-diffusion coefficients of water molecules in four electrolytes. 8

Figure S6 ^{1}H DOSY spectra of water molecules in four electrolytes. 9

Figure S7 2D LF-NMR T1-T2 relaxation spectrum of (a) pure water, (b) $ZnBr_2$ and (c) $ZnCl_2$ electrolyte. 10

Figure S8 The radial distribution function between O atom and O atom of water molecule in pure water and four electrolyte systems. 11

Figure S9 HB number between ClO_4^-, SO_4^{2-} anion and water molecules. 12

Figure S10 FTIR measurements of ClO_4^-, and SO_4^{2-} electrolyte with different concentrations. 13

Figure S11 Orientation relaxation autocorrelation function of four electrolytes. 14

Figure S12 Radial distribution function and the mean effective potential energy path of four anion-H atom pairs. The sequence of the energy barrier is SO_4^{2-}>Cl^->Br^->ClO_4^-. 15

Figure S13 Distribution of the angle θ (shown in the inset) between the vector connecting the anion and the water oxygen atom with the bisector vector of the water molecules. 16

Figure S14 Homemade experimental apparatus used to in-situ detect the freezing points of four electrolytes. 17

Figure S15 DSC test from −75 °C to 0 °C at a heating rate of 5 °C min^{-1} of $Zn(ClO_4)_2$ electrolytes with different concentrations. 18

Figure S16 DSC tests for various Zn based electrolyte with a concentration of 5 m from -80 °C to 10 °C with a heating rate of 5 °C min^{-1}. 19

Figure S17 Representative snapshots of ice crystal growing process in pure water at -20 °C. 20

Figure S18 Representative snapshots of ice crystal growing process in four electrolytes at -20 and -80 °C. 21

Figure S19 (a) The probability distributions of tetrahedral order parameter Q_{tet} for water molecules in pure water and (b) corresponding calculated fraction of ice-like water (tetrahedrality above 0.8) of five systems. 22

Figure S20 (a) Water configuration scheme for better understanding the defined $\boldsymbol{F3}$ and $\boldsymbol{F4}$ parameters. (b-c) The Evolution process of $\boldsymbol{F3}$ and $\boldsymbol{F4}$ parameters during MD simulations at 253 K for $ZnSO_4$ electrolyte (b) and $Zn(ClO_4)_2$ electrolyte (c). 23

Figure S21 (a) Interaction energy between Zn^{2+} and H_2O, (b) RDFs, (c), (d) coordination

number between Zn^{2+} and O atom from H_2O in several electrolyte systems with different anions. 25
Figure S22 Radial distribution function of cation-anion ion pair in four electrolytes. 26
Table S1 The tetrahedral entropy value of pure water and four electrolytes at room temperature. 27
Figure S23 In situ optical microscopic observations of $Zn(NO_3)_2$, ZnI_2 and $Zn(TFSI)_2$ electrolytes before and after freezing. The orange scale bar inside the rightest picture represents 1 mm. 28
Figure S24 The tetrahedral entropy value of seven Zn^{2+}-based electrolytes at respective freezing points. 29
Figure S25 The probability distributions of tetrahedral order parameter Q_{tet} for water molecules in $Zn(ClO_4)_2$ electrolytes with different concentration at their corresponding T_f. 30
Figure S26 DSC tests for various Zn based electrolyte with concentrations of 1 m (left) and 3 m (right) from -80 °C to 10 °C with a heating rate of 5 °C min^{-1}. 31
Figure S27 The tetrahedral entropy value of seven Zn^{2+}-based electrolytes with concentrations of 1 m (left) and 3 m (right) at respective freezing points. 32
Figure S28 The ionic conductivities of the five different electrolytes in the temperature range of -80~+25 °C. 33
Figure S29 The simulated MSD curves and calculated self-diffusion coefficient results of the anions and cations for the five kinds of electrolyte all with the concentration of 5 m under (a)-(c) 223 K and (d)-(f) 300 K. 34
Figure S30 Comparison of the ionic conductivity between several typical aqueous electrolytes for low-temperature batteries reported previously and this work. 35
Figure S31 The configurations and the charge/discharge mechanism of PANI||Zn full batteries. 36
Figure S32 (a) The charge-discharge curves of the PANI||Zn full batteries at 1 A g^{-1} in varying temperature from 25 °C to -70 °C. (b) The optical photograph of the assembled three pouch cells in series trapped in ice and the lighted LEDs. 37
Figure S33 Low-temperature performance comparison between this work and other previous research focusing on other batteries. Notes: the specific capacity of ZIBs in this work was calculated using the mass loading of active materials for cathodes 38
Figure S34 (a)The optical photographs of our fabricated TENG. (b) Transferred charge. (c) Voltage tested by using a high-voltage probe of 500 MΩ. (d) Short-circuit current. 39
Reference 39

Supplementary Figures

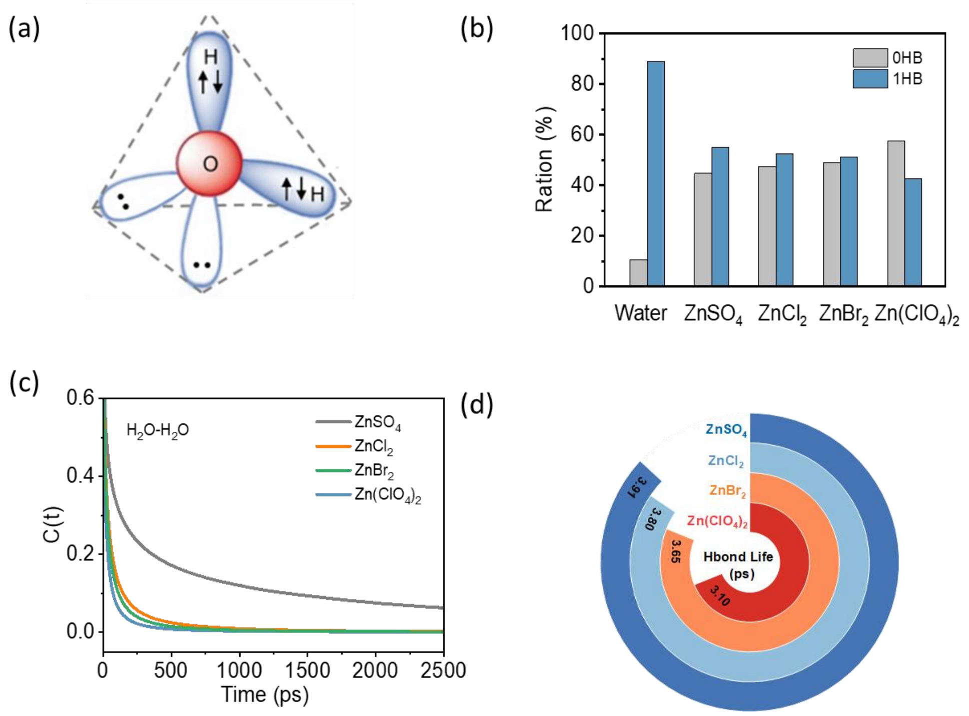

Figure S1 (a) The electronic structure of water molecule. (b)The proportion of water molecules without or with one HB in pure water and four electrolytes at 300K. (c)The HB autocorrelation function C(t) and (d) HB lifetime between water molecules in four electrolytes at 300K.

Figure S1b shows the proportion of water molecules without or with one HB in pure water and four electrolytes. Most water molecules (~90%) forms one hydrogen bonds in pure water. As for four electrolytes, the order of water molecules with one hydrogen bonds follows the sequence of ClO_4^-<Br^-<Cl^-<SO_4^{2-}. The HB autocorrelation function C(t) (Figure S1c) can be obtained by averaging the autocorrelation functions of the existence functions of all HB, which reflects the probability of the time t that HB can retain, as defined as:

$$C(t) = \langle s_i(t_0) s_i(t_0 + t) \rangle_{t_{0,i}} \quad , \quad (1)$$

where s_i(t) reflects the existence of i HB at time t, 0/1= non-exist/exist. And the HB lifetime can be obtained by:

$$\tau_{HB} = \int_0^\infty C(t)\ dt \quad (2)$$

As shown in Figure S1d, the HB lifetime of four electrolytes follows the order of ClO_4^- <Br^-<Cl^-<SO_4^{2-}, verifying the structure-breaking characteristic of ClO_4^-. Highly ordered HB network is hard to form in ClO_4^--based electrolyte, thus the HB between water and water molecules is unstable and owns a shorter lifetime.

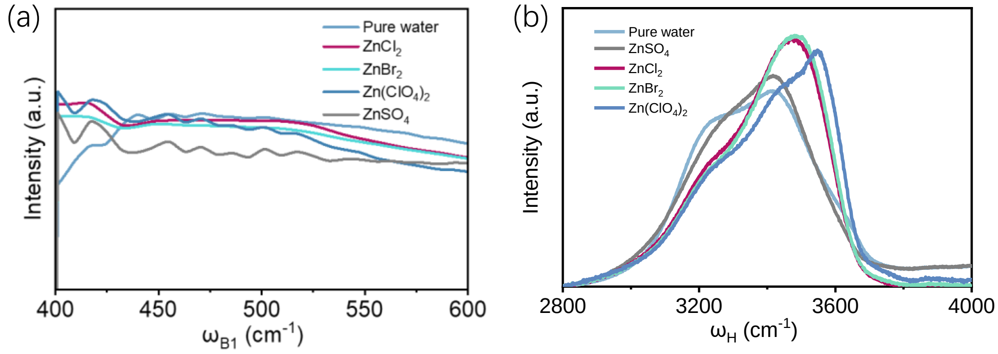

Figure S2 (a) FTIR spectra in the range of 400~600 cm^{-1} and (b)Raman spectra in the range of 2800~4000 cm^{-1}.

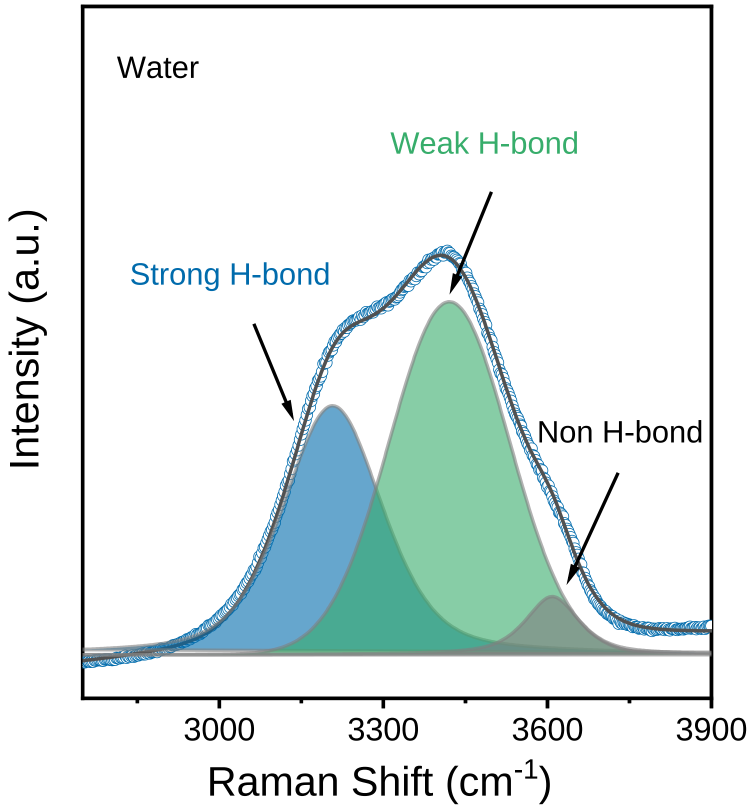

Figure S3 Raman spectroscopy of O–H stretching vibration in pure water, which can be divided into three parts (water molecules with strong HB, weak HB and non-HB).

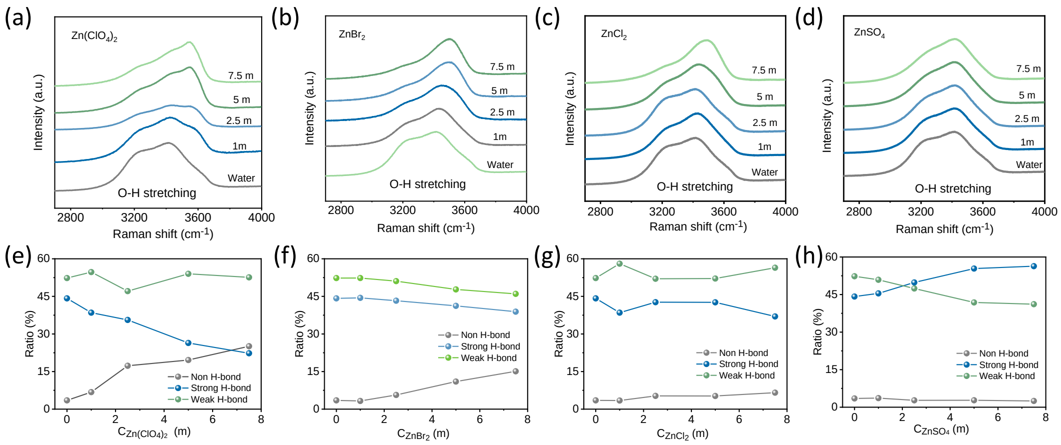

Figure S4 (a-d) Raman spectroscopy of O–H stretching vibration in four electrolytes with different concentrations. (e-h) The proportion of water with strong HB, weak HB and non-HB in four electrolytes with different concentrations.

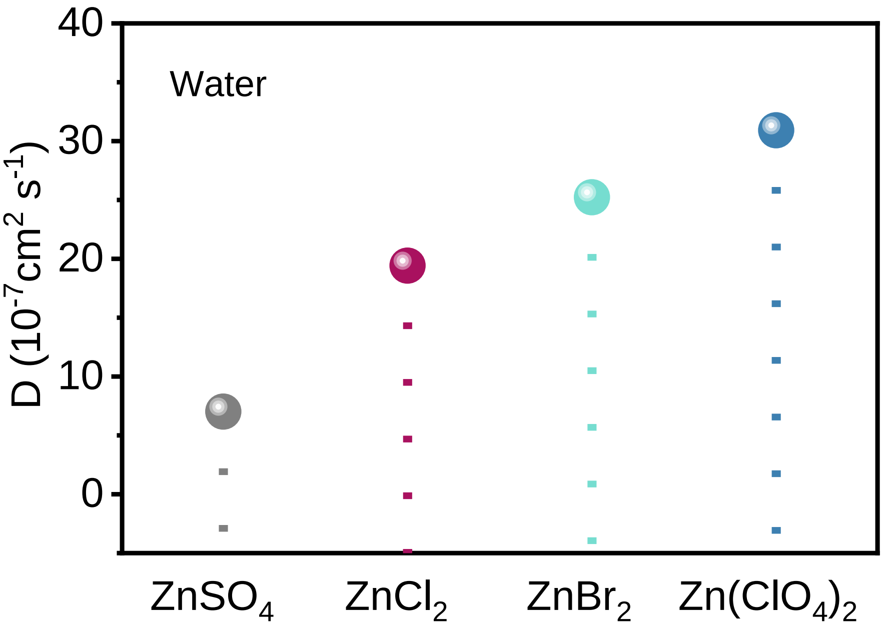

Figure S5 Self-diffusion coefficients of water molecules in four electrolytes.

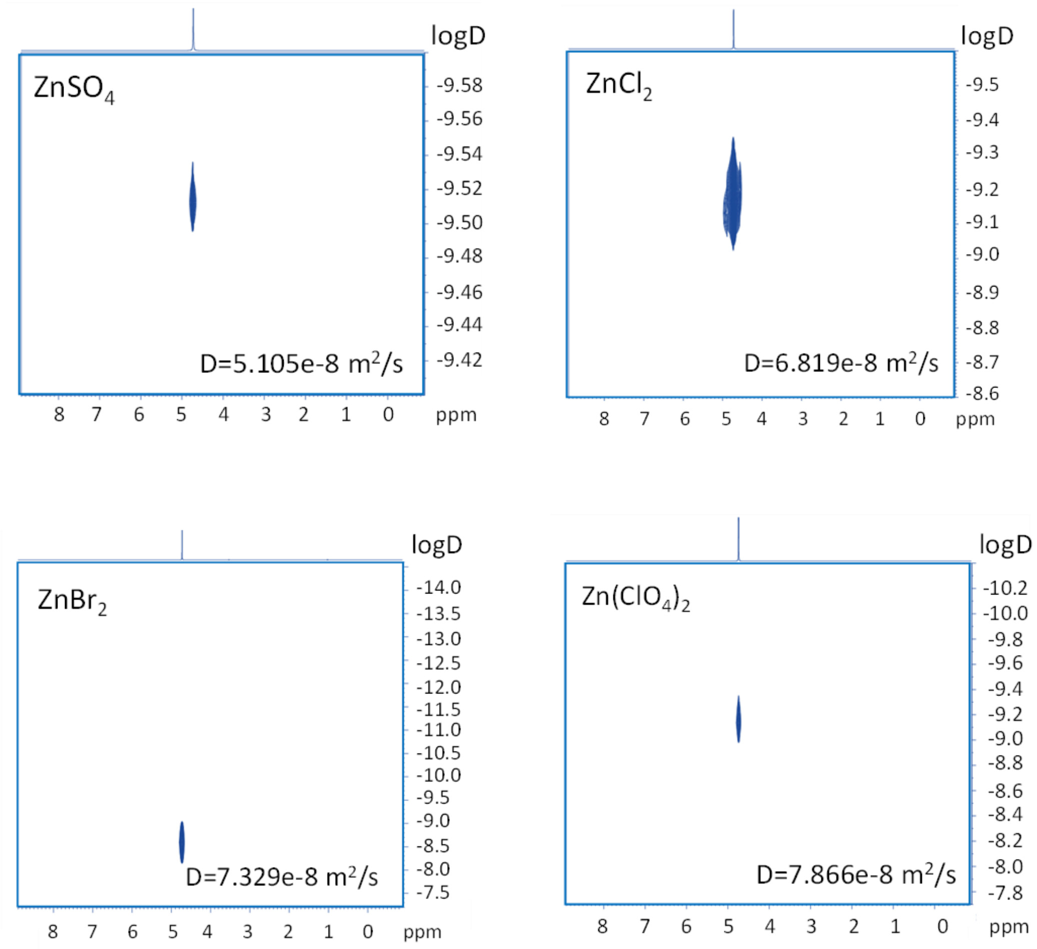

Figure S6 ^{1}H DOSY spectra of water molecules in four electrolytes.

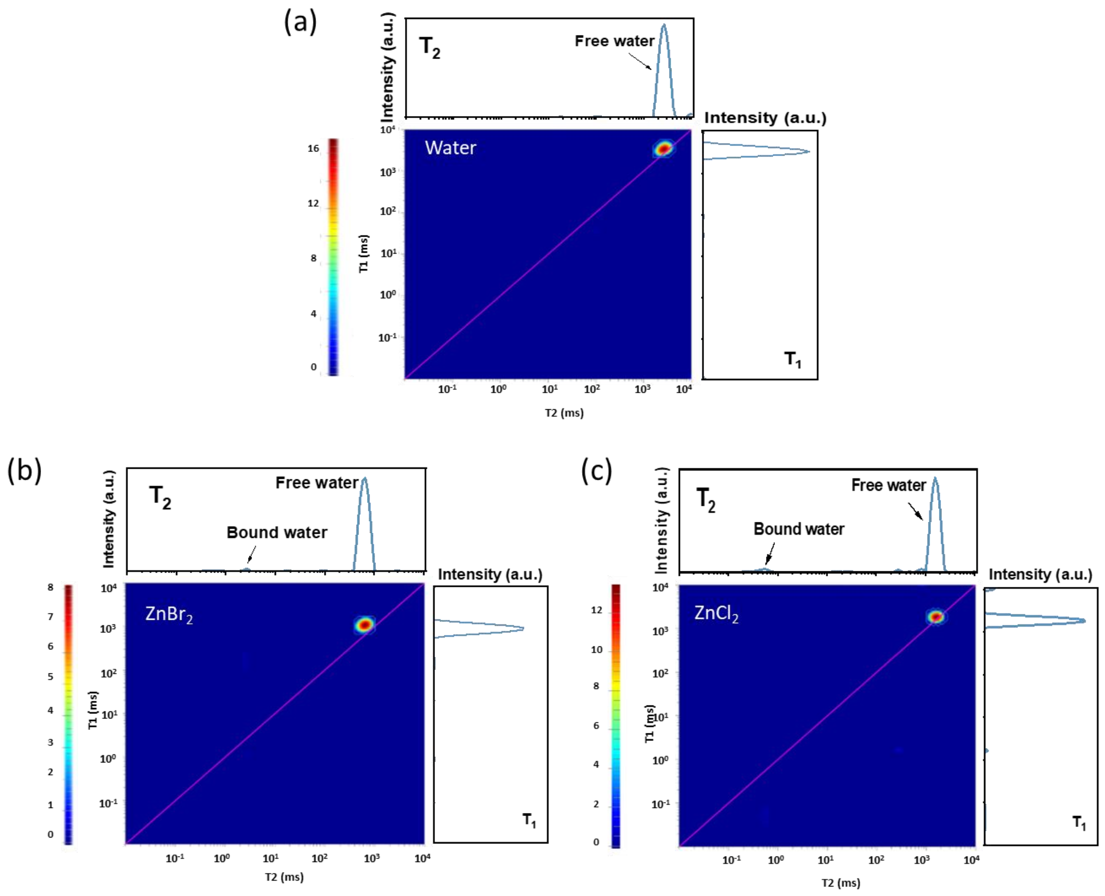

Figure S7 2D LF-NMR T1-T2 relaxation spectrum of (a) pure water, (b) $ZnBr_2$ and (c) $ZnCl_2$ electrolyte.

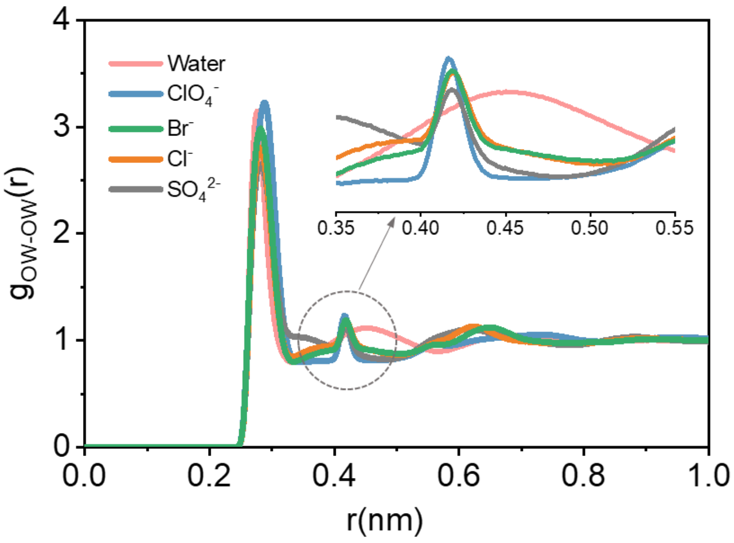

Figure S8 The radial distribution function between O atom and O atom of water molecule in pure water and four electrolyte systems.

The water-water correlation in real space can be described by the oxygen-oxygen (O-O) radical distribution function (RDF) $g_{ow\text{-}ow}(r)$ in Figure S8. In pure water, the second coordination shell captures the tetrahedral geometry with a characteristic peak at ~0.45 nm[8]. Comparing to pure water, the second coordination shells in four electrolytes all become narrower and suffer from an inwards movement, meaning a more compact and highly distorted tetrahedral structure of water networks. The order of the shift degree follows the sequence of ClO_4^->Br^->Cl^->SO_4^{2-}, which coincides well with the ability to destroy the HB network of these four anions.

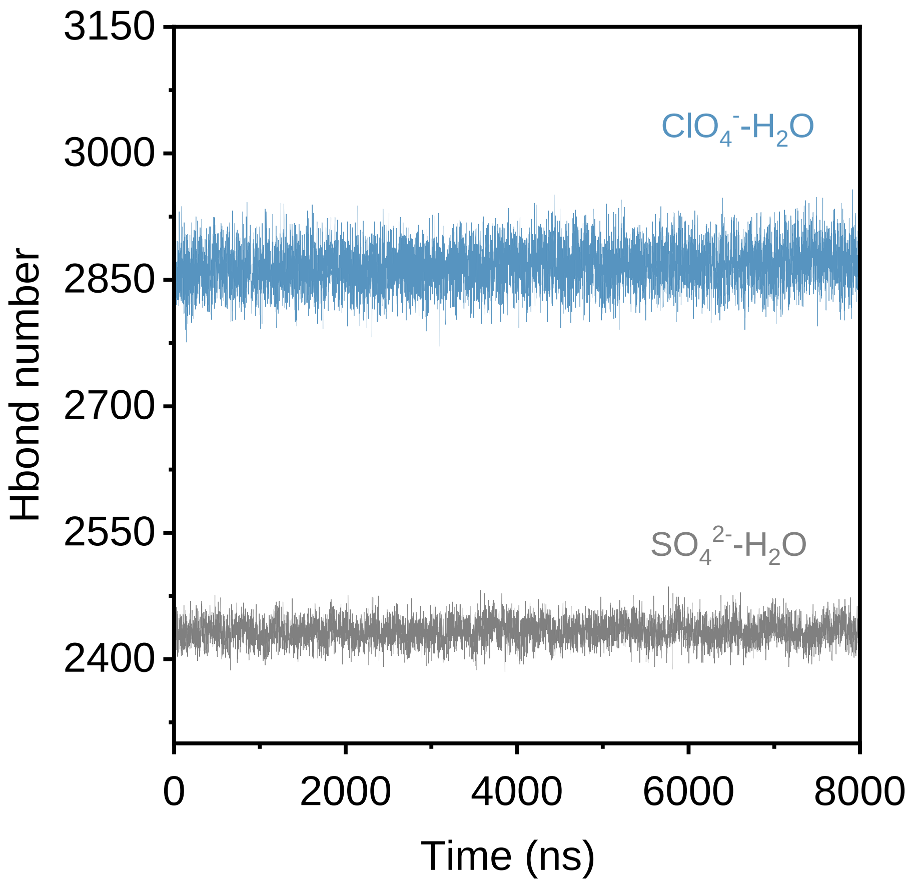

Figure S9 HB number between ClO_4^-, SO_4^{2-} anion and water molecules.

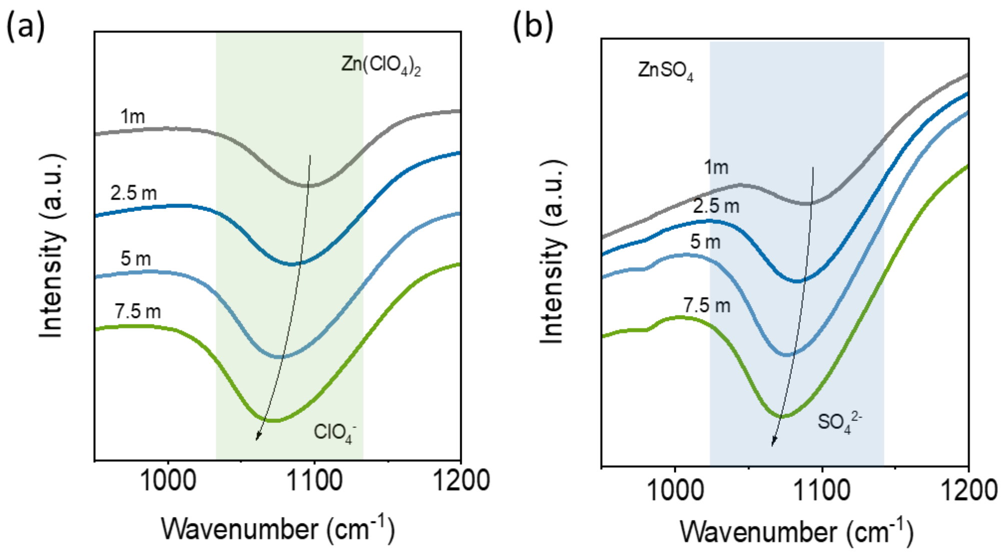

Figure S10 FTIR measurements of ClO_4^-, and SO_4^{2-} electrolyte with different concentrations.

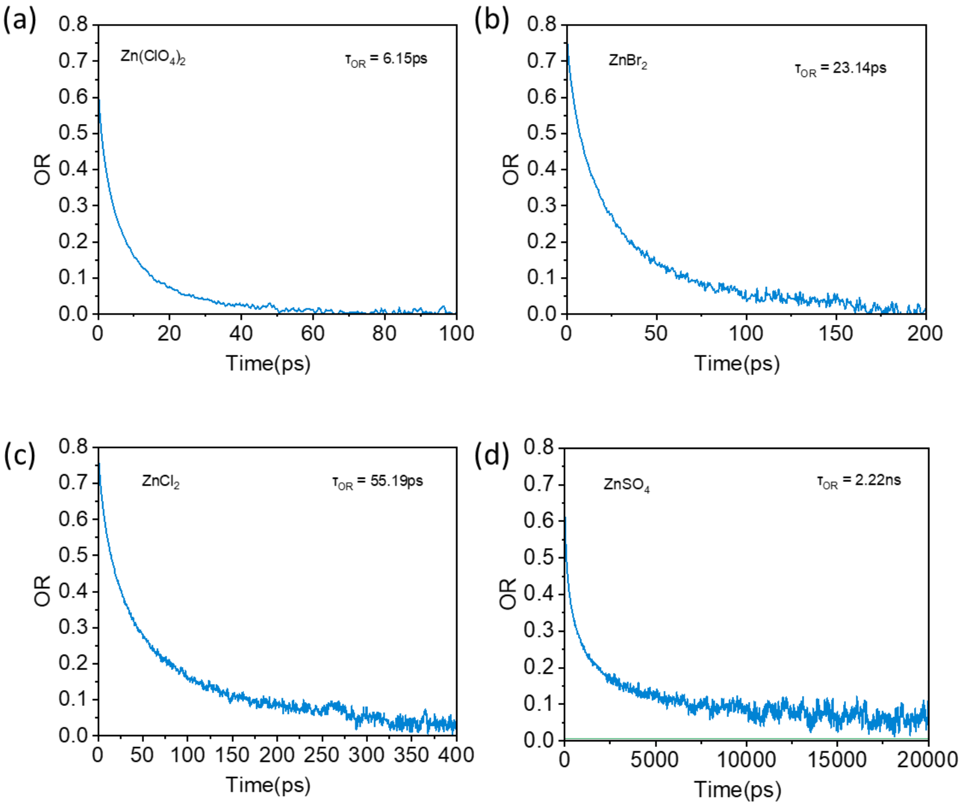

Figure S11 Orientation relaxation autocorrelation function of four electrolytes.

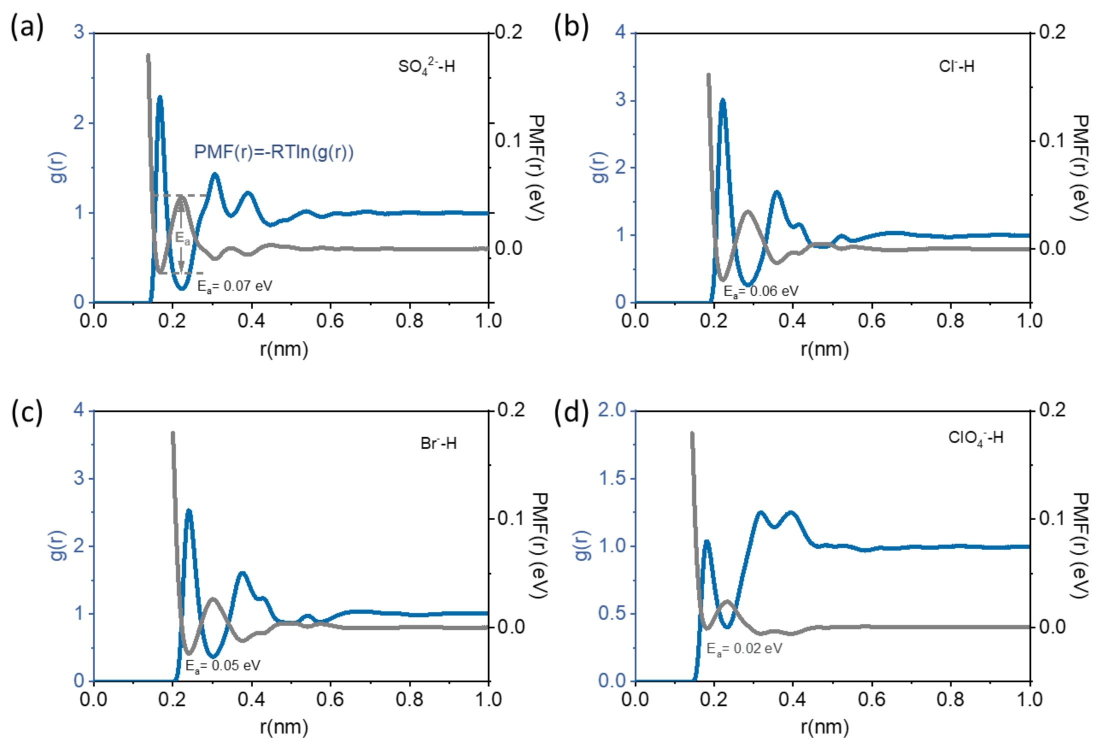

Figure S12 Radial distribution function and the mean effective potential energy path of four anion-H atom pairs. The sequence of the energy barrier is SO_4^{2-}>Cl^->Br^->ClO_4^-.

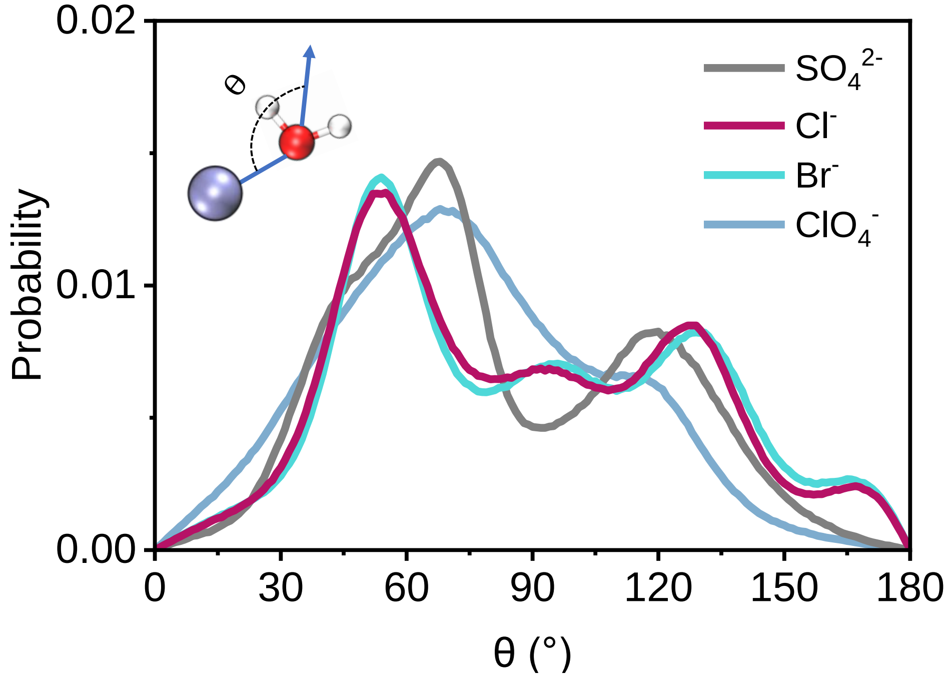

Figure S13 Distribution of the angle θ (shown in the inset) between the vector connecting the anion and the water oxygen atom with the bisector vector of the water molecules.

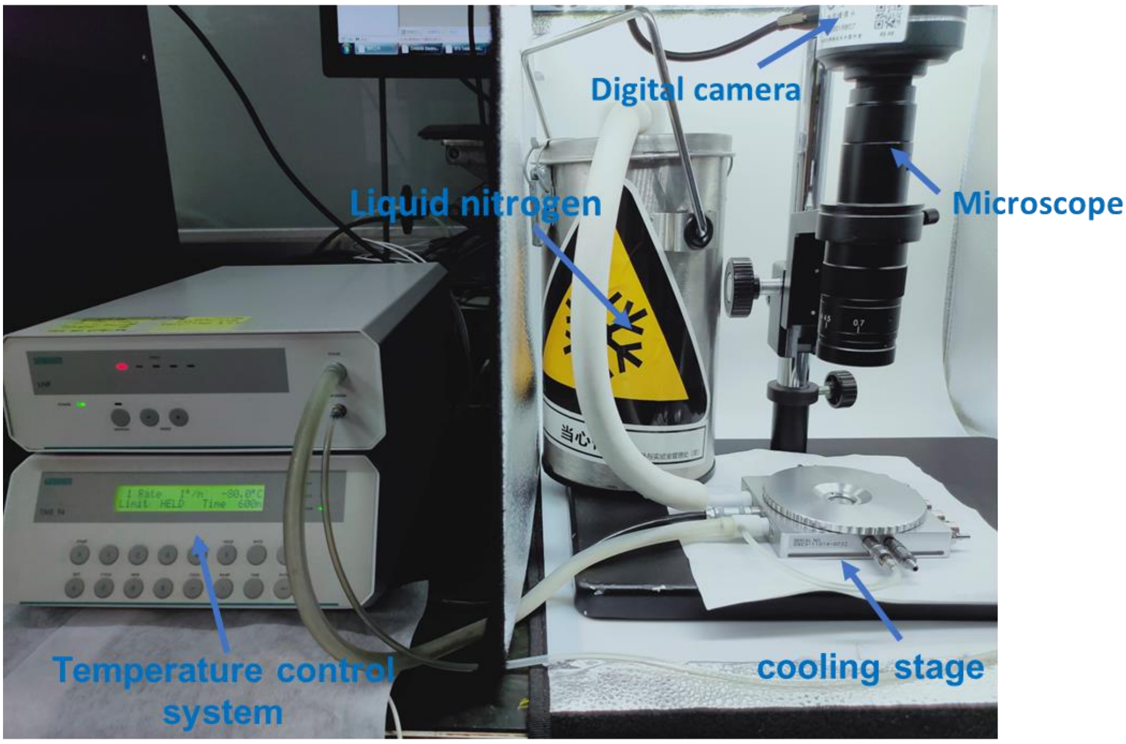

Figure S14 Homemade experimental apparatus used to in-situ detect the freezing points of four electrolytes.

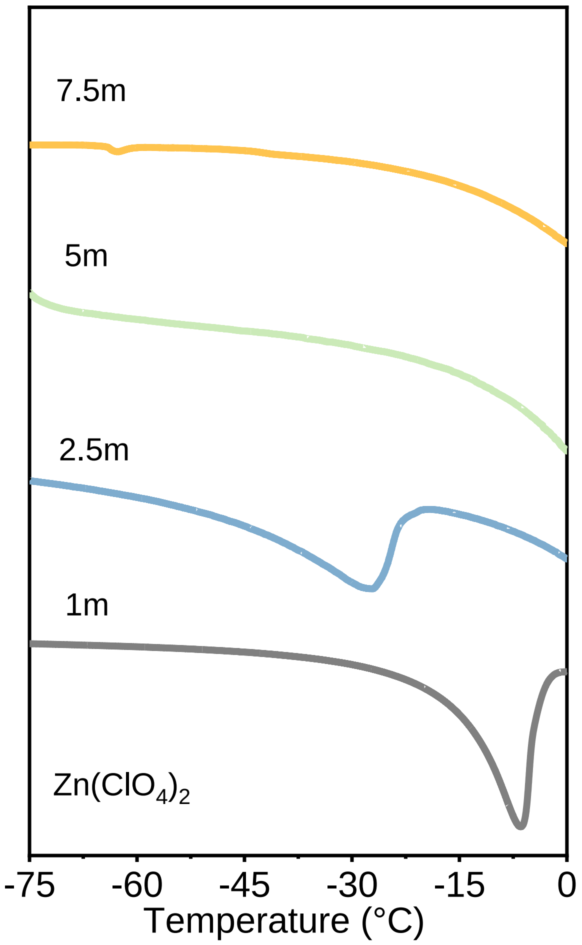

Figure S15 DSC test from −75 °C to 0 °C at a heating rate of 5 °C min^{-1} of $Zn(ClO_4)_2$ electrolytes with different concentrations.

Different scanning calorimeter (DSC) test can specifically reveal the thermodynamic change accompanied by temperature. Figure S15 displays the heat changes of $Zn(ClO_4)_2$ electrolytes with different concentrations by rising the temperature from -70 to 0 °C. The solid-liquid transition and glass-liquid transition point located at different temperatures with the change of the concentration. The ice melting processes generally demonstrates sharp endothermic peaks while the glass-liquid transition displays a step due to the increased heat capacity[9]. It clearly shows that 5 m $Zn(ClO_4)_2$ electrolytes owns lowest freezing point as no endothermic peak and step appear in the temperature range of -70 °C to 0 °C. Therefore, we fixed the concentration of the four salts to 5m for all comparison.

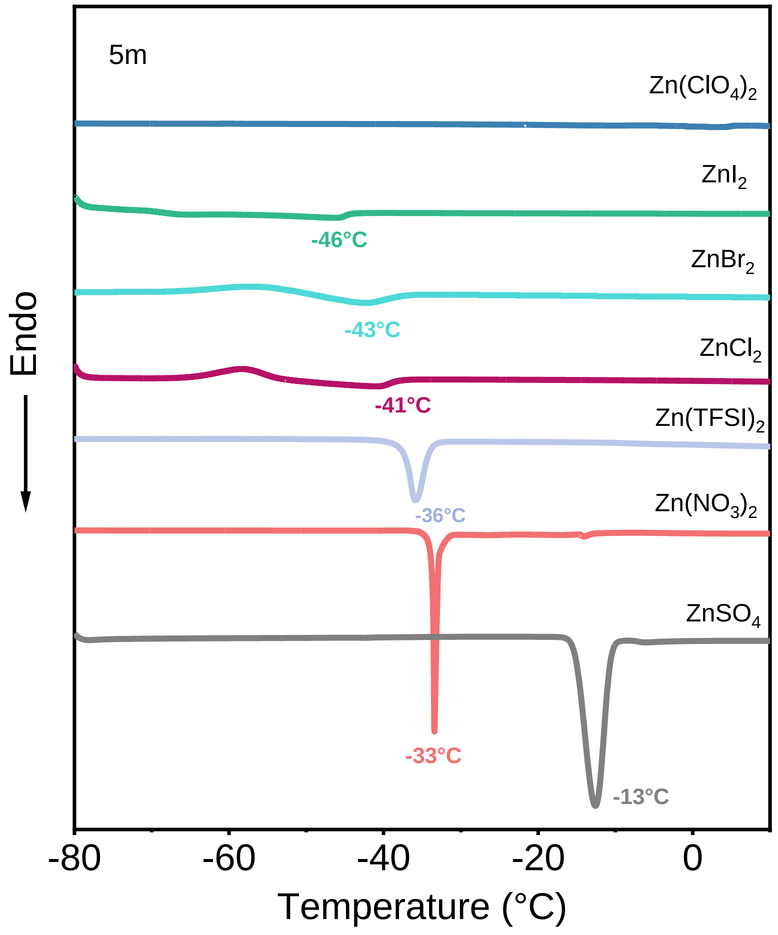

Figure S16 DSC tests for various Zn based electrolyte with a concentration of 5 m from -80 °C to 10 °C with a heating rate of 5 °C min^{-1}.

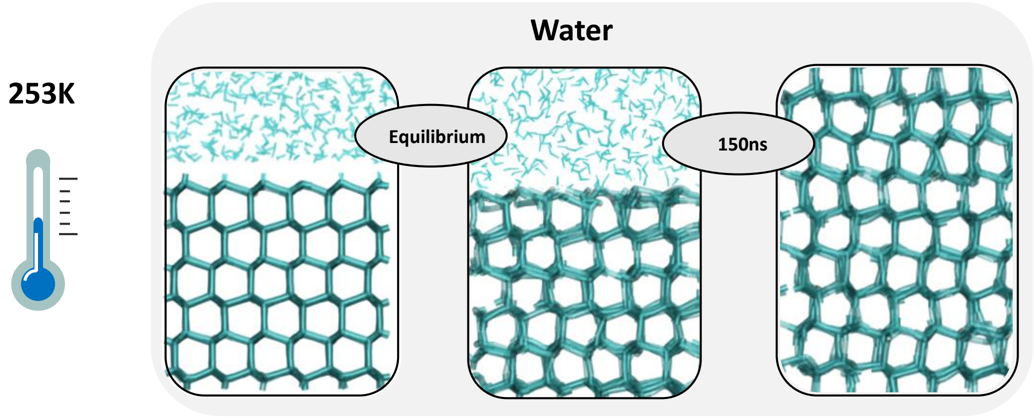

Figure S17 Representative snapshots of ice crystal growing process in pure water at -20 °C.

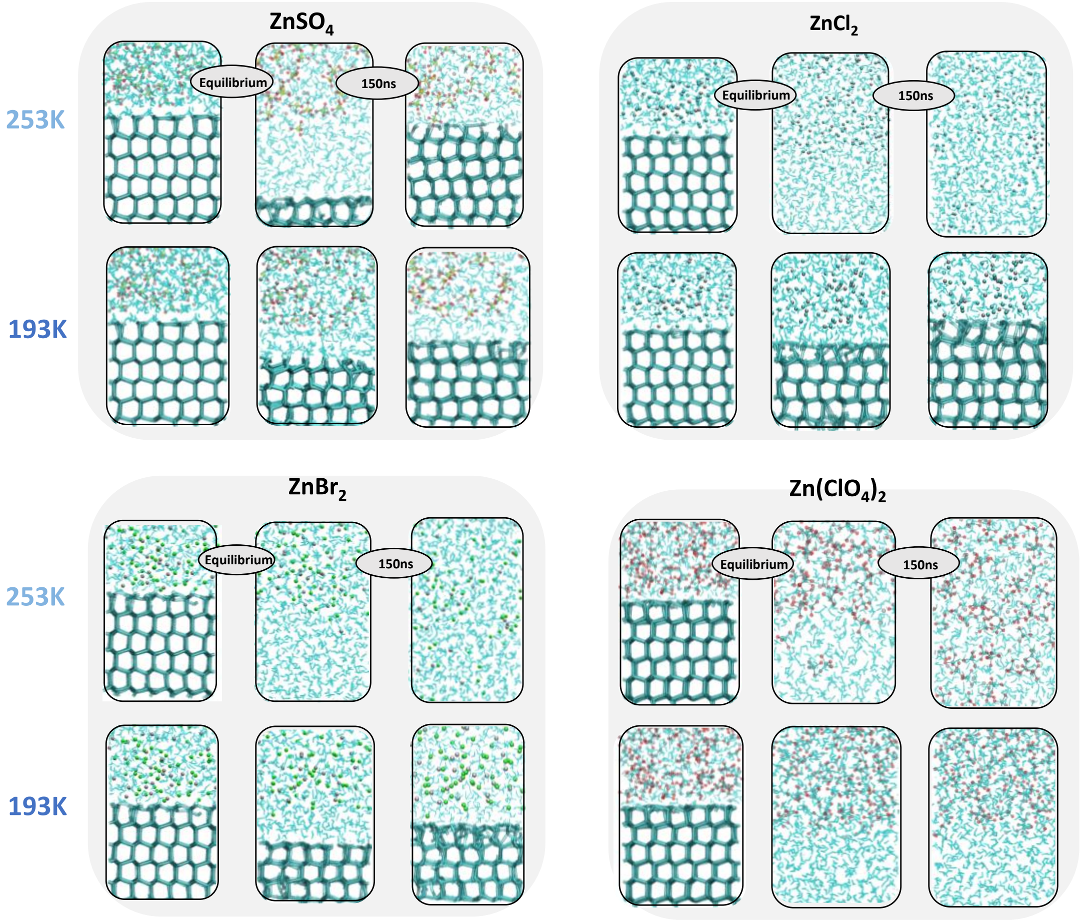

Figure S18 Representative snapshots of ice crystal growing process in four electrolytes at -20 and -80 °C.

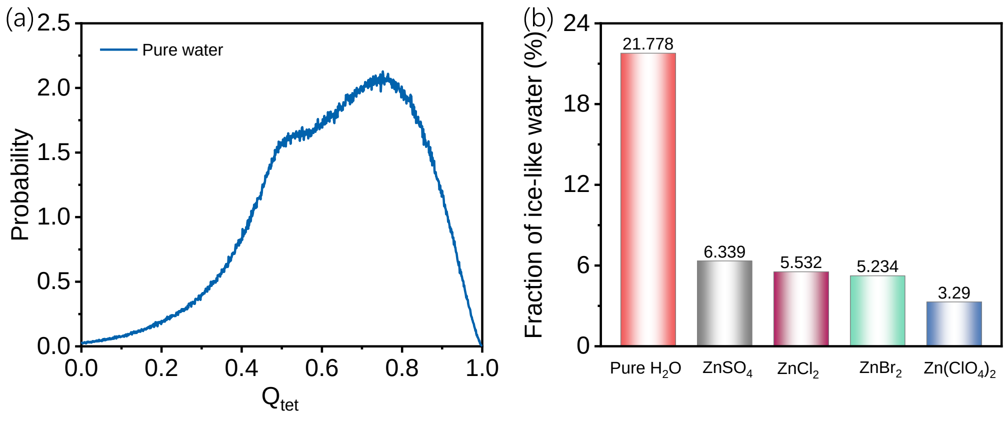

Figure S19 (a) The probability distributions of tetrahedral order parameter Q_{tet} for water molecules in pure water and (b) corresponding calculated fraction of ice-like water (tetrahedrality above 0.8) of five systems.

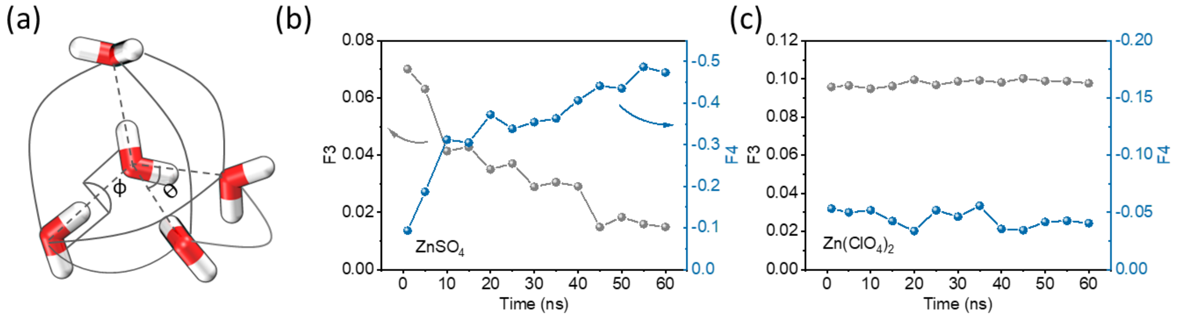

Figure S20 (a) Water configuration scheme for better understanding the defined ***F*3** and ***F*4** parameters. (b-c) The Evolution process of ***F*3** and ***F*4** parameters during MD simulations at 253 K for $ZnSO_4$ electrolyte (b) and $Zn(ClO_4)_2$ electrolyte (c).

The tetrahedral order parameter $F3$ is a parameter composed of a three-body (three water molecules) configuration[10],

$$F3 = \frac{1}{n_i(n_i-1)/2}\sum_{j=1}^{n_{i-1}}\sum_{k=j+1}^{n_i}\left(\left|\cos\theta_{jik}\right|\cos\theta_{jik} + \cos^2(109.47°)\right)^2 , \qquad (3)$$

which characterizes the deviation between the tetrahedron formed by the central oxygen atom and other oxygen atoms in the surrounding 3.5 Å range and the regular tetrahedron. The angle θ involved is the angle between the oxygen atom i of the central water molecule and the oxygen atoms j and k of any two other water molecules within the range of 3.5 Å, of which 109.47 ° is the included angle between the center of the regular tetrahedron and the vertex line. The average values of $F3$ are 0.10 in liquid water and 0.01 in solid water (including ice and hydrate). Four-body order parameter $F4$ is defined as:

$$F4 = \frac{1}{n}\sum_{i=1}^{n}\cos 3\,\Phi_i \qquad (4)$$

, which calculates the dihedral angle Φ formed by the outermost hydrogen atom of two adjacent water molecules and the oxygen atom in water molecules in the system. The average values of $F4$ are -0.04, -0.4 in liquid water and ice, respectively.

Figure S20b and 20c shows the evolution process of $F3$ and $F4$ parameters during MD

simulations at 253 K for SO_4^{2-}-based and ClO_4^- -based electrolyte. Initially, the $F3$ is close to 0.10 and $F4$ is near -0.04 in both systems, indicating the liquid state. After 60-ns simulation, the $F3$ decreases to ~0.01 and $F4$ decreases to ~-0.4 in SO_4^{2-}-based electrolyte, implying the formation of ice crystals. However, in ClO_4^- -based electrolyte system, neither $F3$ nor $F4$ suffers from significant change, suggesting no freezing process occur.

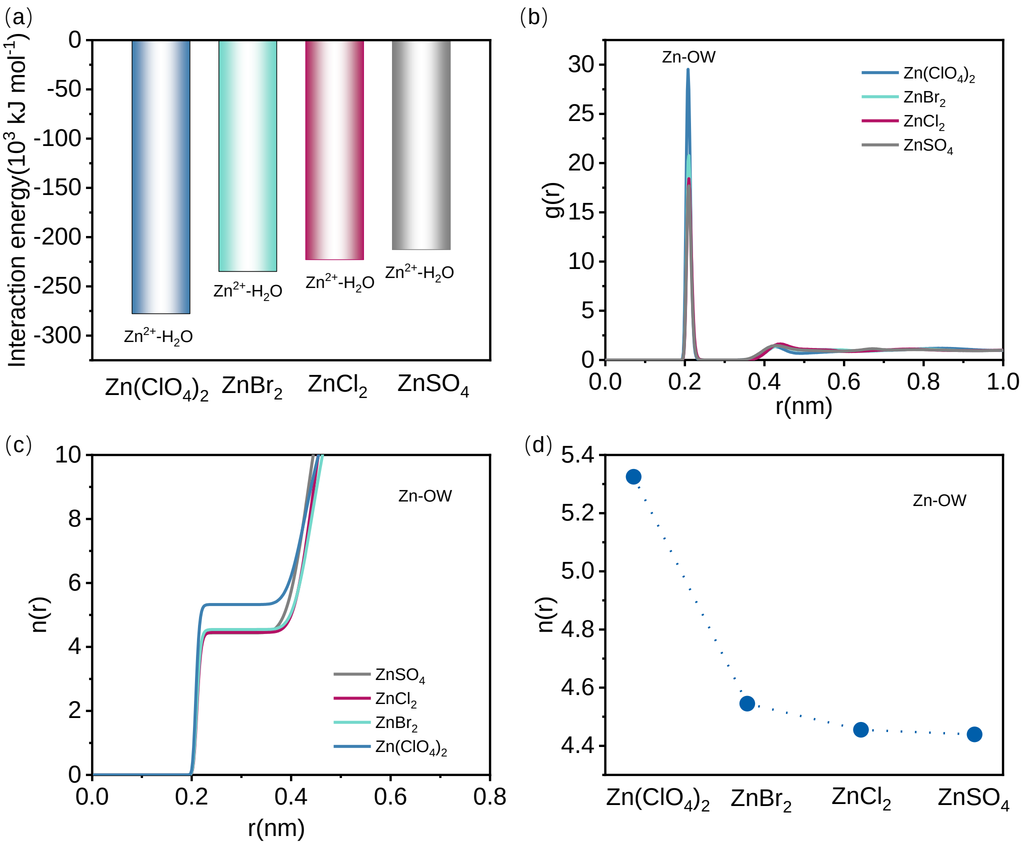

Figure S21 (a) Interaction energy between Zn^{2+} and H_2O, (b) RDFs, (c), (d) coordination number between Zn^{2+} and O atom from H_2O in several electrolyte systems with different anions.

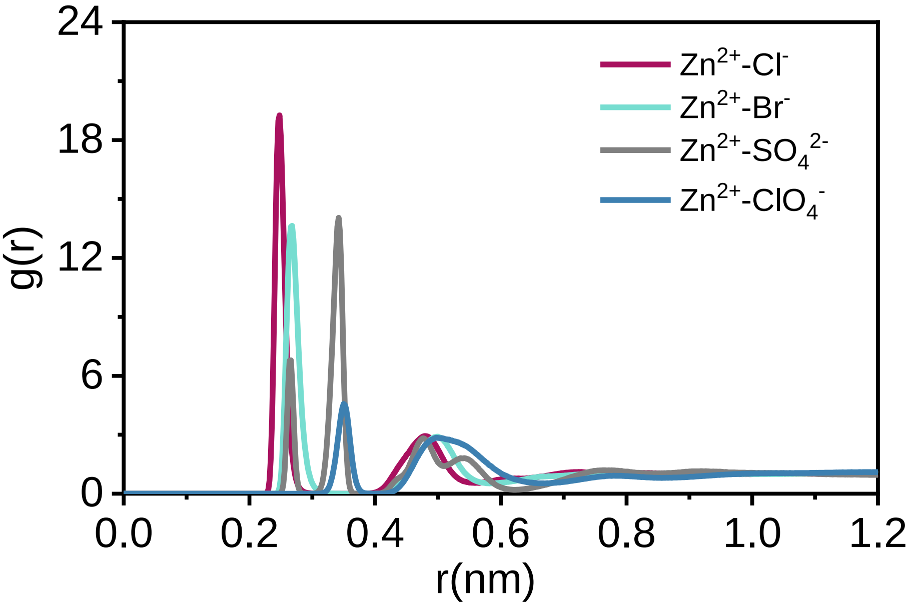

Figure S22 Radial distribution function of cation-anion ion pair in four electrolytes.

Table S1 The tetrahedral entropy value of pure water and four electrolytes at room temperature.

	$(S_Q\text{-}S_0)/k_B$
Water	-1.770
SO_4^{2-}	-1.030
Cl^-	-0.954
Br^-	-0.908
ClO_4^-	-0.726

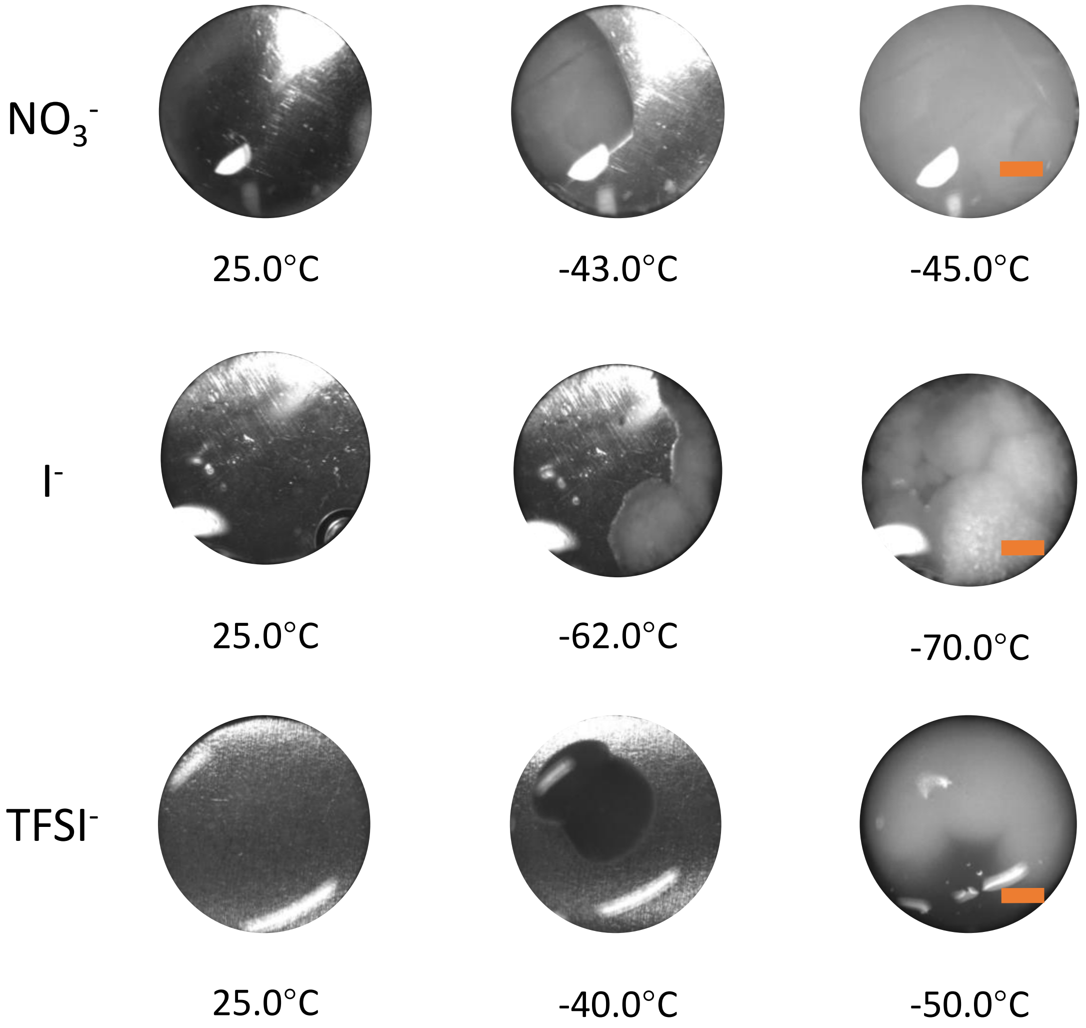

Figure S23 In situ optical microscopic observations of $Zn(NO_3)_2$, ZnI_2 and $Zn(TFSI)_2$ electrolytes before and after freezing. The orange scale bar inside the rightest picture represents 1 mm.

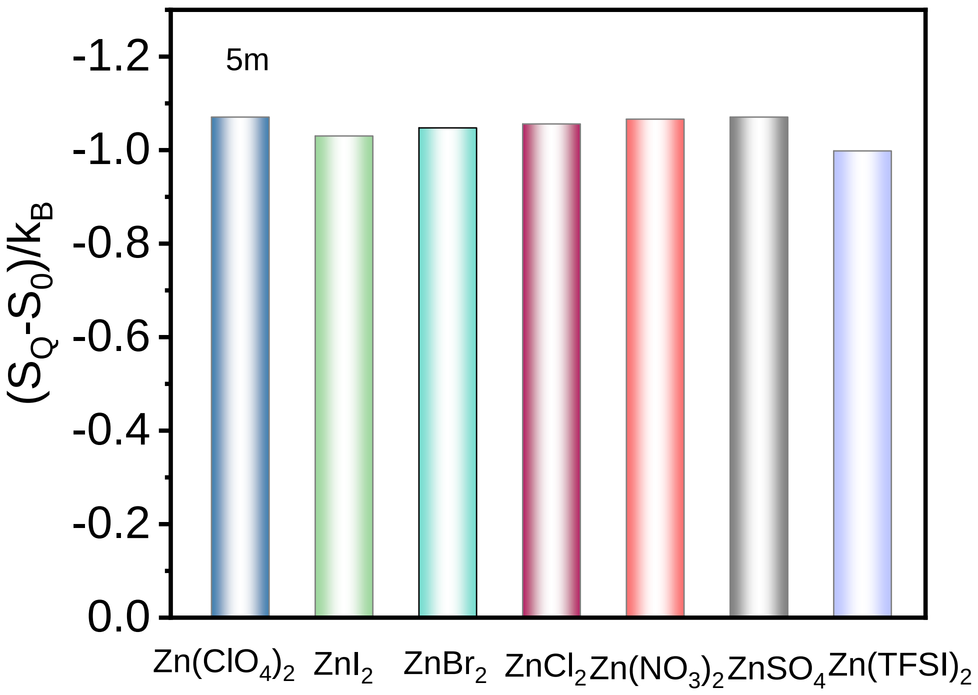

Figure S24 The tetrahedral entropy value of seven Zn^{2+}-based electrolytes at respective freezing points.

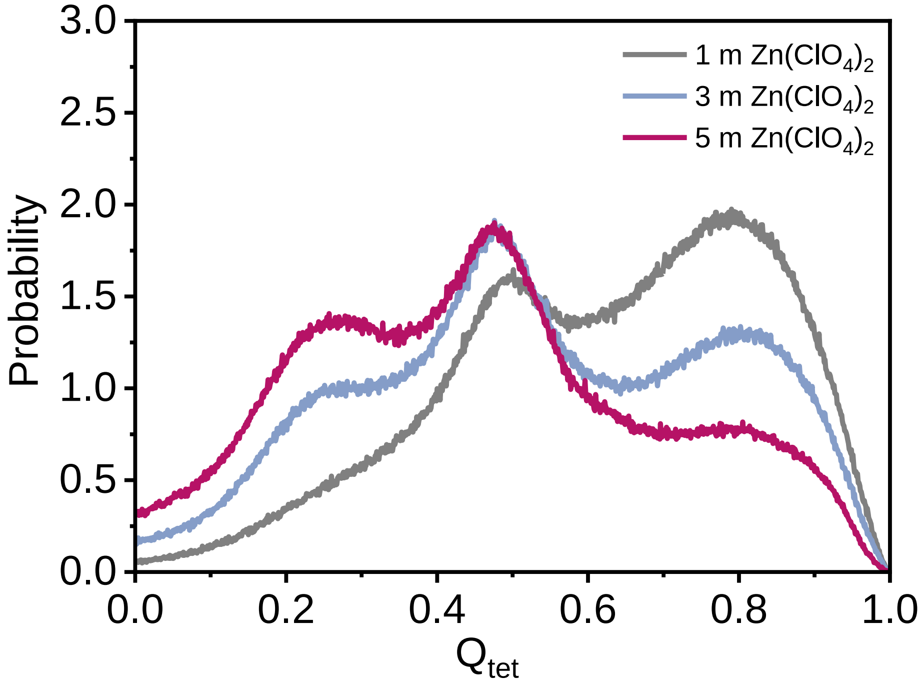

Figure S25 The probability distributions of tetrahedral order parameter Q_{tet} for water molecules in $Zn(ClO_4)_2$ electrolytes with different concentration at their corresponding T_f.

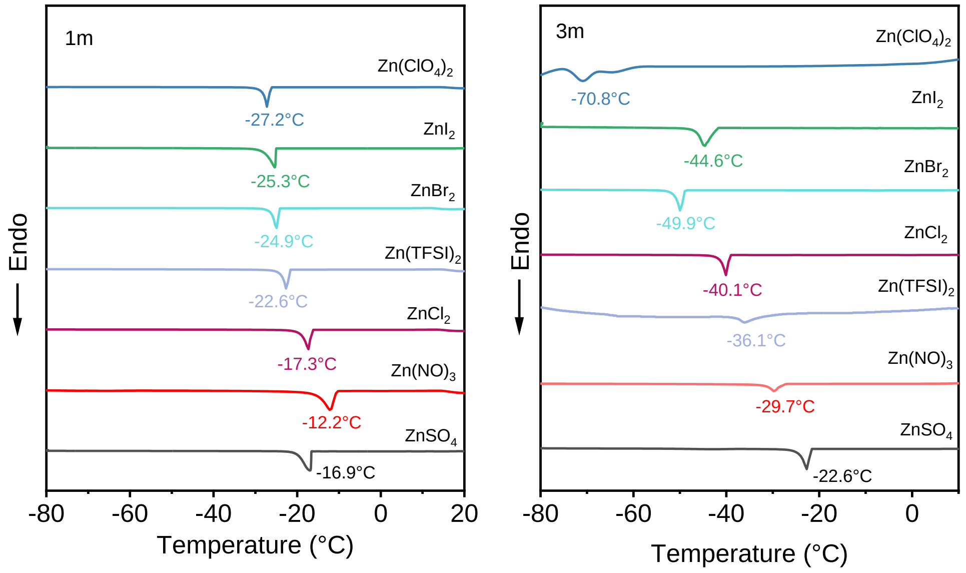

Figure S26 DSC tests for various Zn based electrolyte with concentrations of 1 m (left) and 3 m (right) from -80 °C to 10 °C with a heating rate of 5 °C min^{-1}.

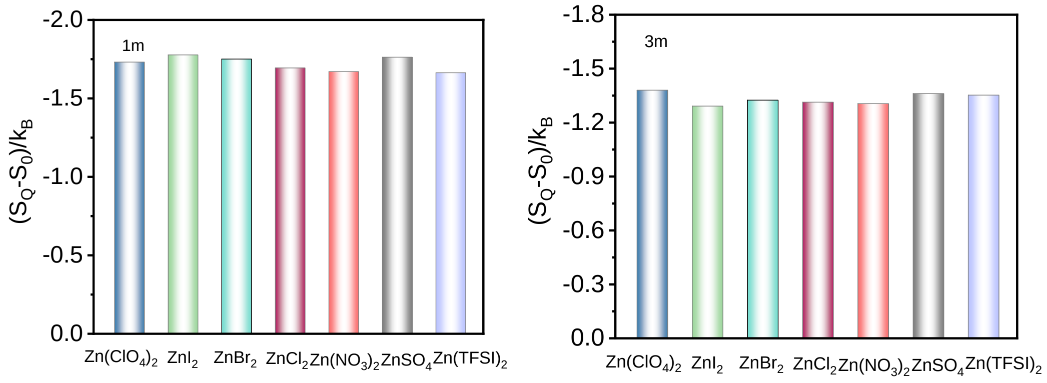

Figure S27 The tetrahedral entropy value of seven Zn^{2+}-based electrolytes with concentrations of 1 m (left) and 3 m (right) at respective freezing points.

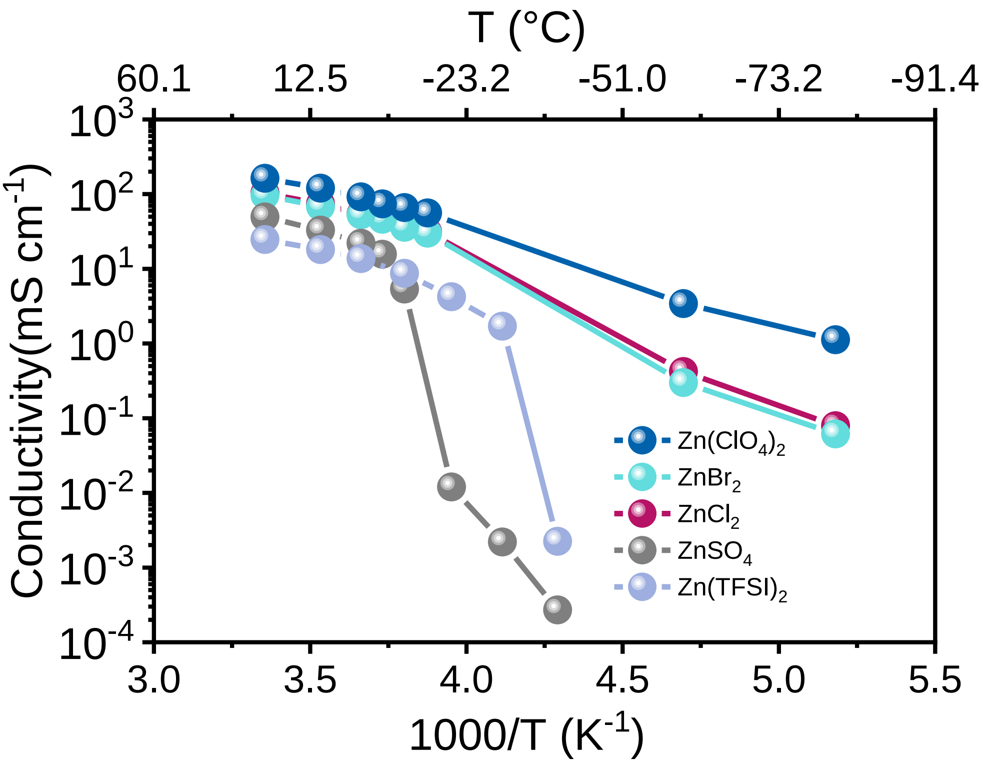

Figure S28 The ionic conductivities of the five different electrolytes in the temperature range of -80~+25 °C.

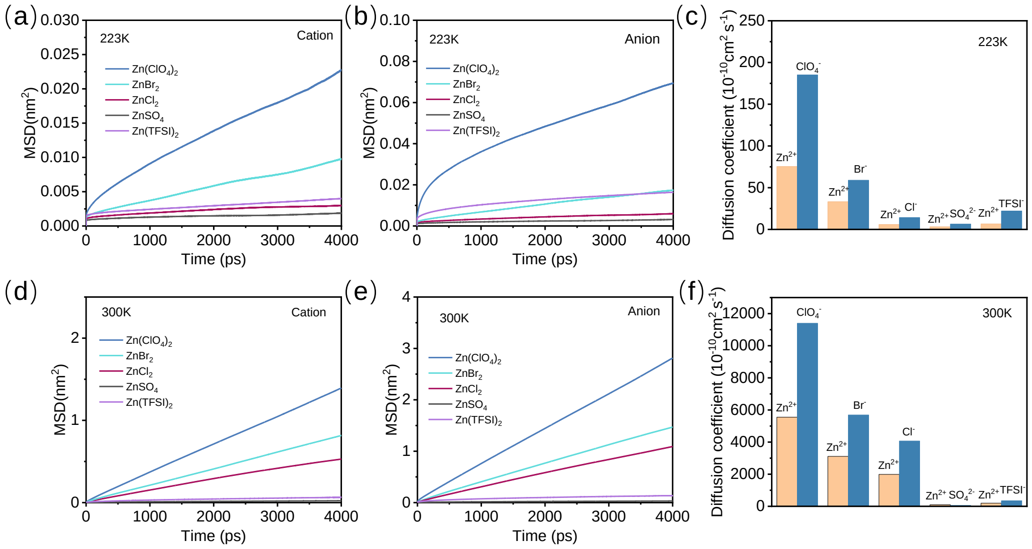

Figure S29 The simulated MSD curves and calculated self-diffusion coefficient results of the anions and cations for the five kinds of electrolyte all with the concentration of 5 m under (a)-(c) 223 K and (d)-(f) 300 K.

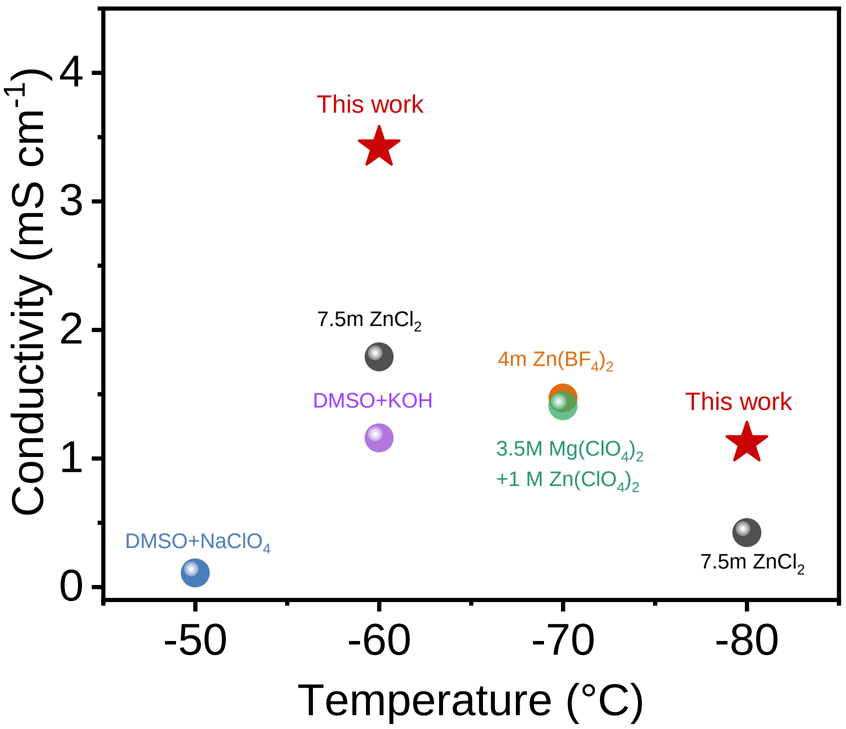

Figure S30 Comparison of the ionic conductivity between several typical aqueous electrolytes for low-temperature batteries reported previously and this work.

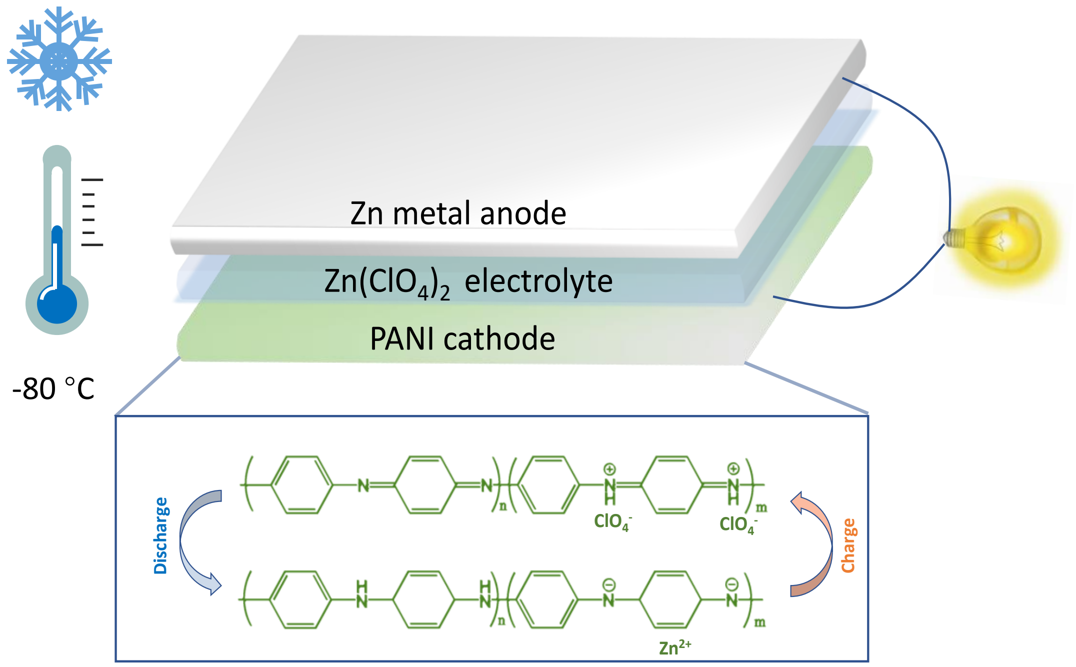

Figure S31 The configurations and the charge/discharge mechanism of PANI||Zn full batteries.

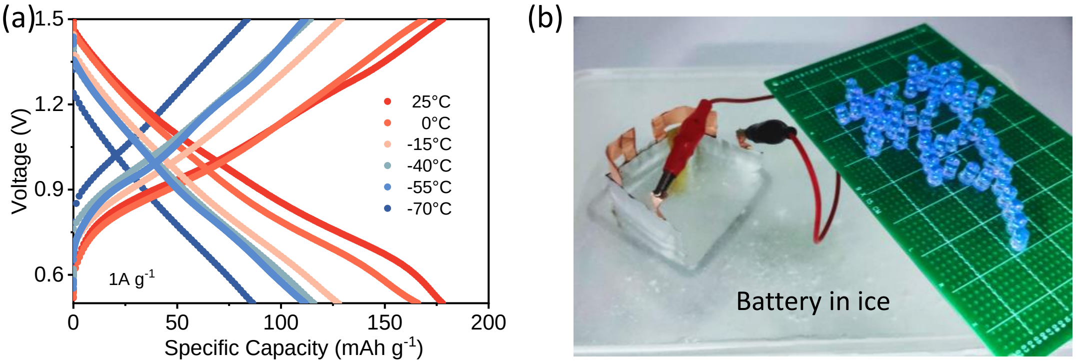

Figure S32 (a) The charge-discharge curves of the PANI||Zn full batteries at 1 A g^{-1} in varying temperature from 25 °C to -70 °C. (b) The optical photograph of the assembled three pouch cells in series trapped in ice and the lighted LEDs.

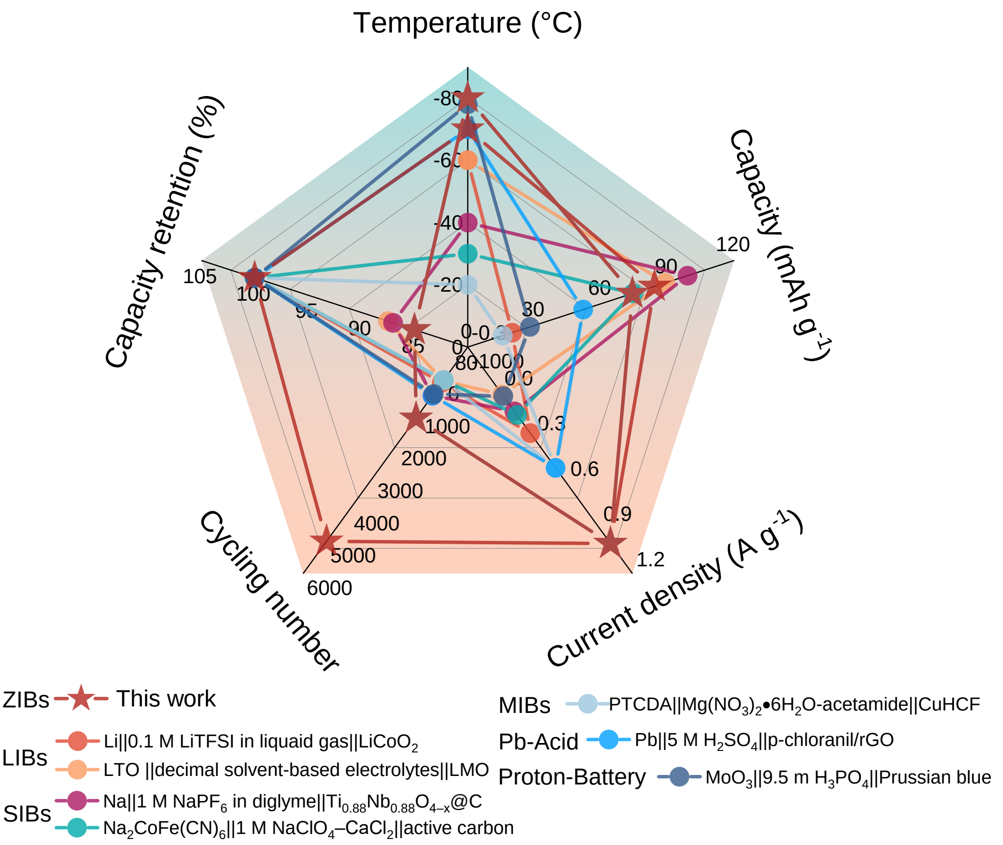

Figure S33 Low-temperature performance comparison between this work and other previous research focusing on other batteries. Notes: the specific capacity of ZIBs in this work was calculated using the mass loading of active materials for cathodes

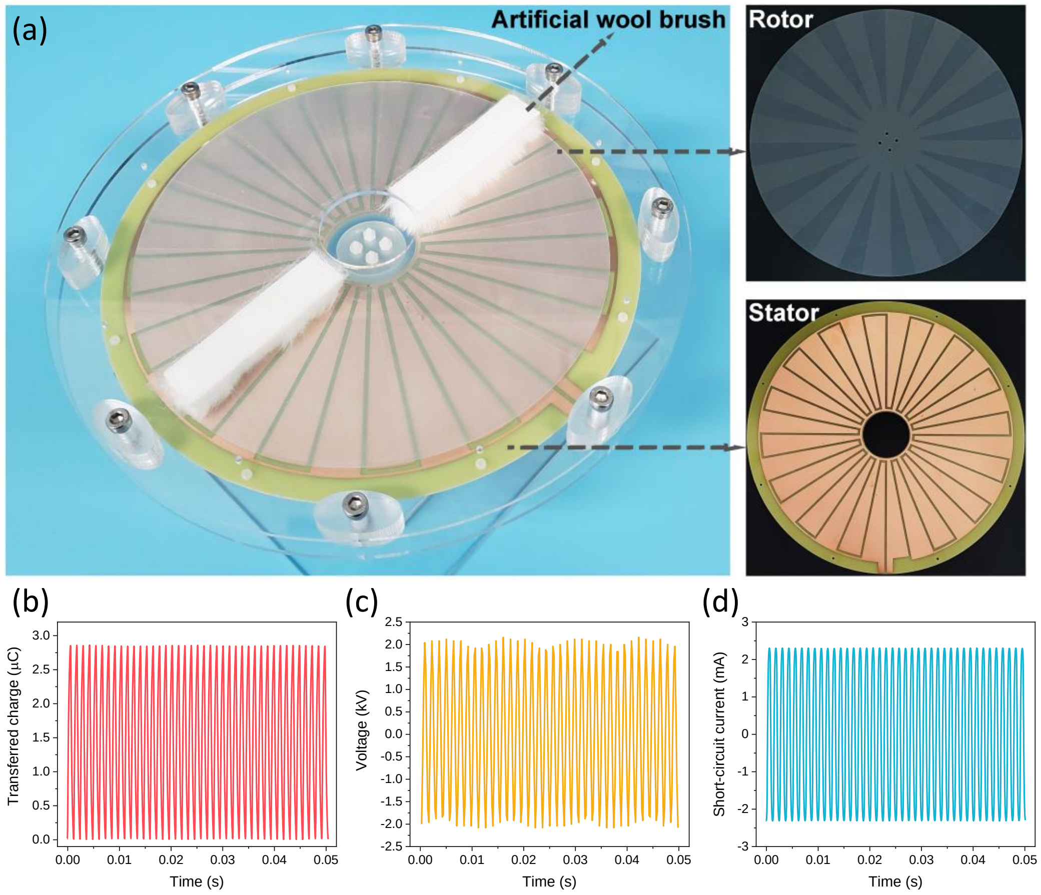

Figure S34 (a)The optical photographs of our fabricated TENG. (b) Transferred charge. (c) Voltage tested by using a high-voltage probe of 500 MΩ. (d) Short-circuit current.

The performance measurements of TENG

With a rotation speed of ~3000 rpm, the basic performance of the TENG at room temperature including transferred charge, voltage with a high-voltage probe, and short-circuit current can achieved 2.8 μC, 2.1 kV, and 2.3 mA, respectively.

Reference

1. Wan, F., *et al.* An Aqueous Rechargeable Zinc-Organic Battery with Hybrid Mechanism. *Adv. Funct. Mater.* **28**, 1804975 (2018).

2. Berendsen, H.J.C., van der Spoel, D. & van Drunen, R. GROMACS: A message-passing parallel molecular dynamics implementation. *Comput. Phys. Commun.* **91**, 43-

56 (1995).
3. Gingrich, T.R. & Wilson, M. On the Ewald summation of Gaussian charges for the simulation of metallic surfaces. *Chemical Physics Letters* **500**, 178-183 (2010).
4. Bussi, G., Donadio, D. & Parrinello, M. Canonical sampling through velocity rescaling. *J. Chem. Phys.* **126**, 014101 (2007).
5. Izadi, S. & Onufriev, A.V. Accuracy limit of rigid 3-point water models. *J. Chem. Phys.* **145**, 074501 (2016).
6. Li, Z., Song, L.F., Li, P. & Merz, K.M., Jr. Systematic Parametrization of Divalent Metal Ions for the OPC3, OPC, TIP3P-FB, and TIP4P-FB Water Models. *J. Chem. Theory Comput.* **16**, 4429-4442 (2020).
7. Abascal, J.L., Sanz, E., Garcia Fernandez, R. & Vega, C. A potential model for the study of ices and amorphous water: TIP4P/Ice. *J. Chem. Phys.* **122**, 234511 (2005).
8. Zhang, C., Yue, S., Panagiotopoulos, A.Z., Klein, M.L. & Wu, X. Dissolving salt is not equivalent to applying a pressure on water. *Nat. Commun.* **13**, 822 (2022).
9. Verdonck, E., Schaap, K. & Thomas, L.C. A discussion of the principles and applications of Modulated Temperature DSC (MTDSC). *Int. J. Pharmaceut.* **192**, 3-20 (1999).
10. Gao, F., Gupta, K.M., Yuan, S. & Jiang, J. Decomposition of CH4 hydrate: effects of temperature and salt from molecular simulations. *Molecular Simulation* **44**, 1220-1228 (2018).
