# Supplementary material for: Tailoring water structure with high-tetrahedral-entropy for antifreezing electrolytes and energy storage at −80 °C
Source: Nat Commun. 2023 Feb 3;14:601. doi: 10.1038/s41467-023-36198-5 (PMC9898254; doi:10.1038/s41467-023-36198-5)
Supplement: Supplementary file 2 — Description of Additional Supplementary Files [file 41467_2023_36198_MOESM2_ESM.pdf]

Description of Additional Supplementary Files

File Name: Supplementary Movie 1
Description: MD simulations (150 ns) for a liquid-ice interfacial system of 5 m ZnSO4 electrolyte under a temperature of -20 °C

File Name: Supplementary Movie 2
Description: MD simulations (150 ns) for a liquid-ice interfacial system of 5 m $Zn(ClO_4)_2$ electrolyte under a temperature of -20 °C
